# Combined effects of crude oil exposure and warming on eggs and larvae of an arctic forage fish

**DOI:** 10.1038/s41598-021-87932-2

**Published:** 2021-04-16

**Authors:** Morgan Lizabeth Bender, Julia Giebichenstein, Ragnar N. Teisrud, Jennifer Laurent, Marianne Frantzen, James P. Meador, Lisbet Sørensen, Bjørn Henrik Hansen, Helena C. Reinardy, Benjamin Laurel, Jasmine Nahrgang

**Affiliations:** 1 Department of Arctic and Marine Biology, UiT The Arctic University of Norway, 9037 Tromsø, Norway; 2 Akvaplan-Niva, Fram Centre, 9296 Tromsø, Norway; 3 Environmental and Fisheries Sciences Division, Northwest Fisheries Science Center, National Marine Fisheries Service, National Oceanic and Atmospheric Administration, 2725 Montlake Blvd. East, Seattle, Washington, 98112 USA; 4 SINTEF Ocean, Environment and New Resources, 7465 Trondheim, Norway; 5 Scottish Association for Marine Science, Oban, PA37 1QA UK; 6 Department of Arctic Technology, The University Centre in Svalbard, Longyearbyen, Svalbard Norway; 7 Fisheries Behavioral Ecology Program, Alaska Fisheries Science Center, National Marine Fisheries Service, NOAA, Hatfield Marine Science Center, Newport, OR 97365 USA

**Keywords:** Ecophysiology, Embryology, Environmental impact

## Abstract

Climate change, along with environmental pollution, can act synergistically on an organism to amplify adverse effects of exposure. The Arctic is undergoing profound climatic change and an increase in human activity, resulting in a heightened risk of accidental oil spills. Embryos and larvae of polar cod (*Boreogadus saida*), a key Arctic forage fish species, were exposed to low levels of crude oil concurrently with a 2.3 °C increase in water temperature. Here we show synergistic adverse effects of increased temperature and crude oil exposure on early life stages documented by an increased prevalence of malformations and mortality in exposed larvae. The combined effects of these stressors were most prevalent in the first feeding larval stages despite embryonic exposure, highlighting potential long-term consequences of exposure for survival, growth, and reproduction. Our findings suggest that a warmer Arctic with greater human activity will adversely impact early life stages of this circumpolar forage fish.

## Introduction

The Arctic is undergoing profound environmental change with declining sea ice, higher surface temperatures, changes in community structure, and increased human activity^1,2^. Climate change and environmental pollution can act synergistically to challenge an organism or population beyond the degree of any single stressor^3^. Climate change has been seen to amplify the adverse effects of exposure to low levels of pollutants in Arctic wildlife^4^, which may have the potential for population-level effects. The combined effect of climate change and environmental pollution on Arctic organisms and ecosystem is largely unknown but population declines are suggested at environmentally realistic pollution levels in combination with moderate temperature rise by the limited research exploring effects of multiple stressors in the Arctic^4^.

The loss of sea ice and warming is occurring rapidly in many Arctic seas^5–7^. In the Barents Sea, warmer Atlantic water masses (> 2.5 °C) are predicted to replace the cooler Arctic waters (< 1 °C) before the end of the century^5^ and the frequency of low ice and ice-free years is predicted to increase^8^. Concurrent with rising sea surface temperatures, declining sea ice has facilitated an increase in ship traffic in the Arctic by 60% since 2012^9^. With heightened activity comes increased risk of petroleum pollution^10^. Shipping routes (i.e., the Northern Sea Route and Northwest passage route) traverse important nursery grounds for Arctic fish species^11–13^.

Early life stages (ELSs) of many fish face greater vulnerability to environmental stressors, owing to physical and biological factors^14,15^. To ensure survival and a strong year class, pelagic eggs and larvae must maintain their position in the water column through buoyancy control and swim bladder inflation, forage when yolk reserves are exhausted, grow quickly, and build up energy reserves for overwintering while avoiding predation^16^. Fish ELSs are more sensitive than juveniles or adults to temperatures outside their thermal window^14,17^ and toxicants^15,18^ and may present a bottleneck for species facing environmental change. Increased sensitivity is attributed to the relatively underdeveloped organs, lower metabolic scope, and reduced cardiorespiratory capacity during early ontogeny^19–21^, and a large surface area to volume ratio compared to adults^16^. Furthermore, ELSs have limited mobility and a restricted vertical distribution putting them at a higher risk for direct exposure to increased sea surface temperatures and pollution events in surface waters. Most studies investigating the combined effects of temperature and crude oil exposure on ELSs of fish have focused on warm water species, such as Mahi–mahi (*Coryphaena hippurus*), especially in the wake of the Deepwater Gulf Horizon oil spill in 2010^22^.

In light of the environmental change and growing pollution risk in the Arctic and the vulnerability of fish ELSs to environmental stressors, an investigation on the combined effects of the changing Arctic on the success of key species was warranted. Polar cod (*Boreogadus saida*) is an endemic circumpolar Arctic species with a large population biomass, high energy content, high trophic connectivity, and high societal value despite a limited commercial harvest^23–25^. Polar cod are cold-adapted and early life stages are often found in association with sea ice^26,27^. Embryo survival is reduced at temperatures above 3 °C ^17,28^. Polar cod stocks in the Barents Sea, Iceland-East Greenland waters, parts of the Bering Sea, Canadian Arctic Archipelago, and in Disko Bay, Greenland have been in decline or are modeled to be in decline and retreating northward with increased water temperatures^23,29–33^. Climate-driven changes in life-history traits (e.g., size-at-age and individual fecundity) have already been suggested in mature polar cod^34^. Biophysical modeling of Barents Sea polar cod ELS have already observed the northward retreat of spawning locations with temperatures reaching 5.7 °C annually and further forecast an “imminent recruitment collapse” with continued sea-ice reduction and increased warming^17,35^. Previous work on polar cod early life stages exposed to crude oil has revealed lethal effects at low exposure concentrations^36^ and long-term physiological effects in juveniles^37^; however, the combined effects of increased temperature and crude oil exposure are unknown.

Generally, exposure of fish eggs and larvae to increased temperature (i.e., at the top of their thermal window) can increase enzymatic activity, including induction of heat shock proteins which can be quantified using mRNA expression of *hsp* 70 and *hsp* 8, increase metabolism, and accelerate development while decreasing the efficiency of metabolism and reducing the energy available for development^17,38^. These elevated temperatures can also decrease the duration of embryogenesis, reduce size and survival of hatchlings, increase larval growth rate, and increase the frequency of deformities in many fish species^39–41^, including polar cod^26,28^. Exposure to crude oil in developing fish is hypothesized to act through multiple pathways causing developmental toxicity and sublethal effects that persist to later life stages even at very low concentrations^37,42–44^. Crude oil exposure triggered gene pathways involved in biotransformation (e.g., cytochrome P450 (*cyp1a* and *cyp1c1*) mRNA expression), stress response, lipid metabolism, and ion regulation in ELSs of Atlantic haddock (*Melanogrammus aeglefinus*)^45^. On the organismal level, crude oil exposure has resulted in reductions in larval size, defects of the heart, increased incidence of edema and deformities, reduced growth, and reduced survival in many fish species^22,43,46^, including polar cod^36,37^. Greater metabolic demands, higher frequency of deformities, altered cardiac function, and changes in embryo buoyancy were reported as synergistic effects (i.e., greater than the effects of these two stressors combined) of temperature stress and crude oil exposure in Mahi–mahi^47,48^. Increased metabolic costs associated with exposure to elevated temperature may limit energy available for detoxification processes in fish ELSs^49^ and thereby increase the potentially toxic effects of petroleum exposure.

Here, an experiment was conducted where polar cod ELS were exposed to increased temperatures and crude oil pollution at environmentally realistic levels starting after egg fertilization and continuing into exogenous feeding larval stages. Experimental conditions were designed to mimic an Arctic oil spill at three concentrations of the water-soluble fraction (WSF) of crude oil across two thermal conditions, 0.5 °C (present) and 2.8 °C (future, year 2100^5^). We hypothesized that combined exposure to crude oil and warmer water temperatures would act synergistically, leading to more severe morphological and physiological outcomes than any singular stressor. We investigated the responses in polycyclic aromatic hydrocarbon (PAH) bioaccumulation, mRNA expression of biotransformation and stress-related genes, embryo specific gravity, heart rate and arrhythmia, growth, survival, developmental rate, and deformities in polar cod ELSs. Endpoints were compared across crude oil concentrations and temperature groups to ascertain the possible additive, antagonistic, or synergistic effects at embryonic, yolk sac, and feeding larval stages.

## Results

### Crude oil exposure

**Figure 1:**
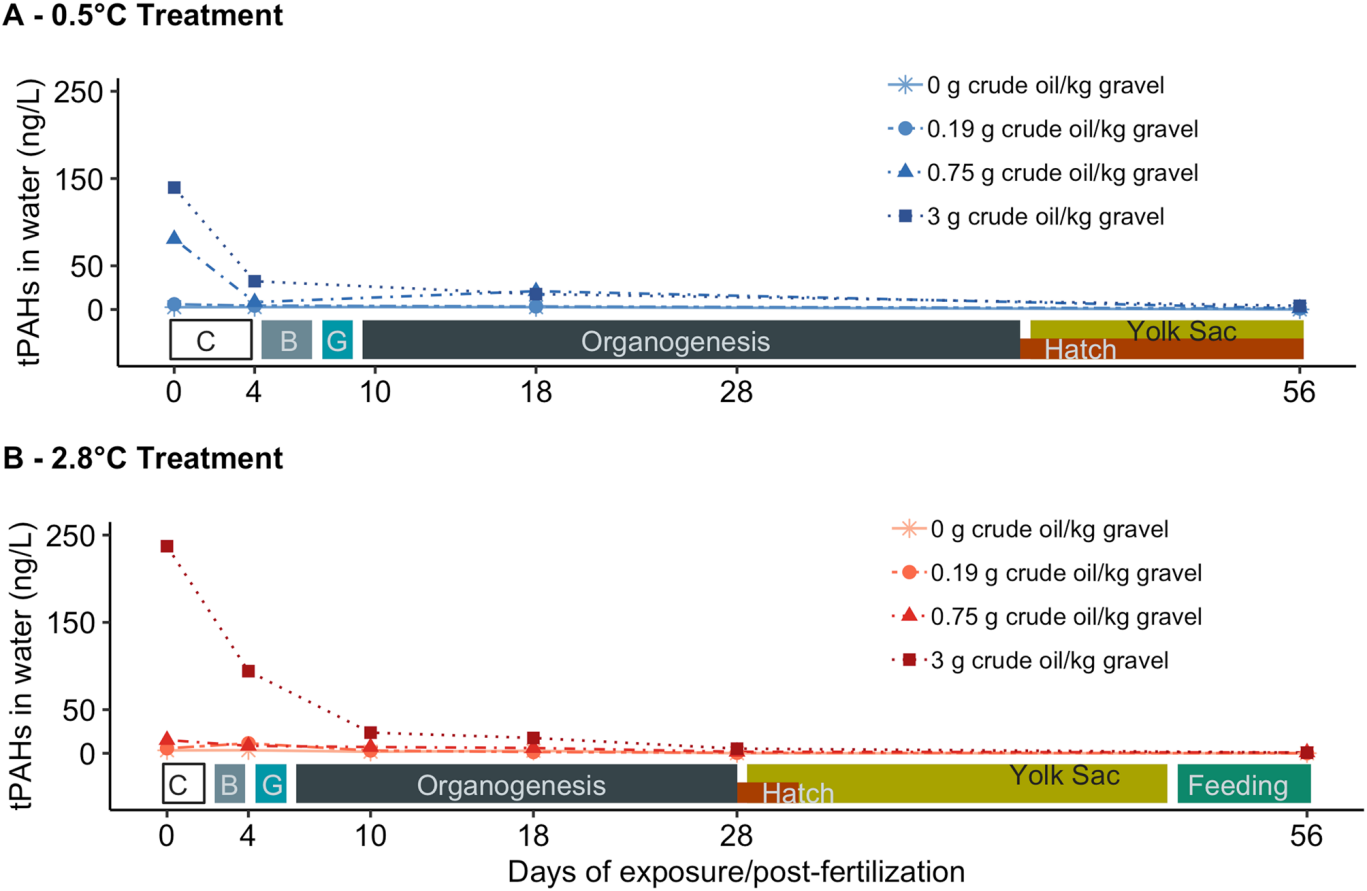
The concentration of tPAHs (ng/L) over time in (**A**) the cold, 0.5 °C temperature group and (**B**) the warm, 2.8 °C temperature group. Different concentrations of crude oil and gravel are distinguished by color, shape and line type. The best linear model *lm(tPAH* ~ *Time* + *factor(Temperature)* + *factor(Oil Treatment)* reported significant effects of time (F-value = 6.09, p-value = 0.019), and oil treatment (F-value = 4.5034, p-value = 0.009), but not temperature (F-value = 0.0211, p-value = 0.885). The developmental timeline of embryos and larvae in each temperature group are displayed in each panel. Early embryogenesis is divided into cleavage stage (C), blastulation (B), and gastrulation (G).

Following the flushing of the oiled rock cylinders and start of the exposure, total PAH (tPAH) concentrations (calculated from 44 parent and alkylated PAHs) were generally low, correlated with crude oil concentration in gravel (r = 0.93, *p* < 0.01), and declined over time (Fig. 1, Supplementary Table S1). The highest concentrations of tPAHs were measured at the start (day 0) in the warm, high oil treatment (237 ng/L tPAHs) followed by the cold, high oil treatment (140 ng/L tPAHs). For the hatching period (day 28 in warm- and day 56 in cold-incubated treatments), all treatments had reached tPAH levels ~ 1 ng/L except for the highest crude oil exposure at both temperature groups (4–5 ng/L tPAHs). While no particulate oil was observed in the effluent nor was any sheen visible on the surface of the incubators, analysis of water samples by comprehensive two-dimensional GCxGC-MS analysis showed a typical crude oil profile in the warm, high exposure effluent at day 0 and day 4. This indicates the presence of 'bulk' oil in the exposure system, likely in the form of microdroplets (Supplementary Fig. S1). In addition to the PAHs and alkylated homologues, several petroleum compound groups were observed in the extracts, including *n*-alkanes, branched and cyclic aliphatics, as well as a complex set of monoaromatic compounds in this mostly qualitative analysis (Supplementary Fig. S1). While steadily decreasing, the ‘bulk’ oil-like profile remained for the first 4 days of exposure. No ‘bulk’ oil profile was observed in the cold treatment.

**Figure 2:**
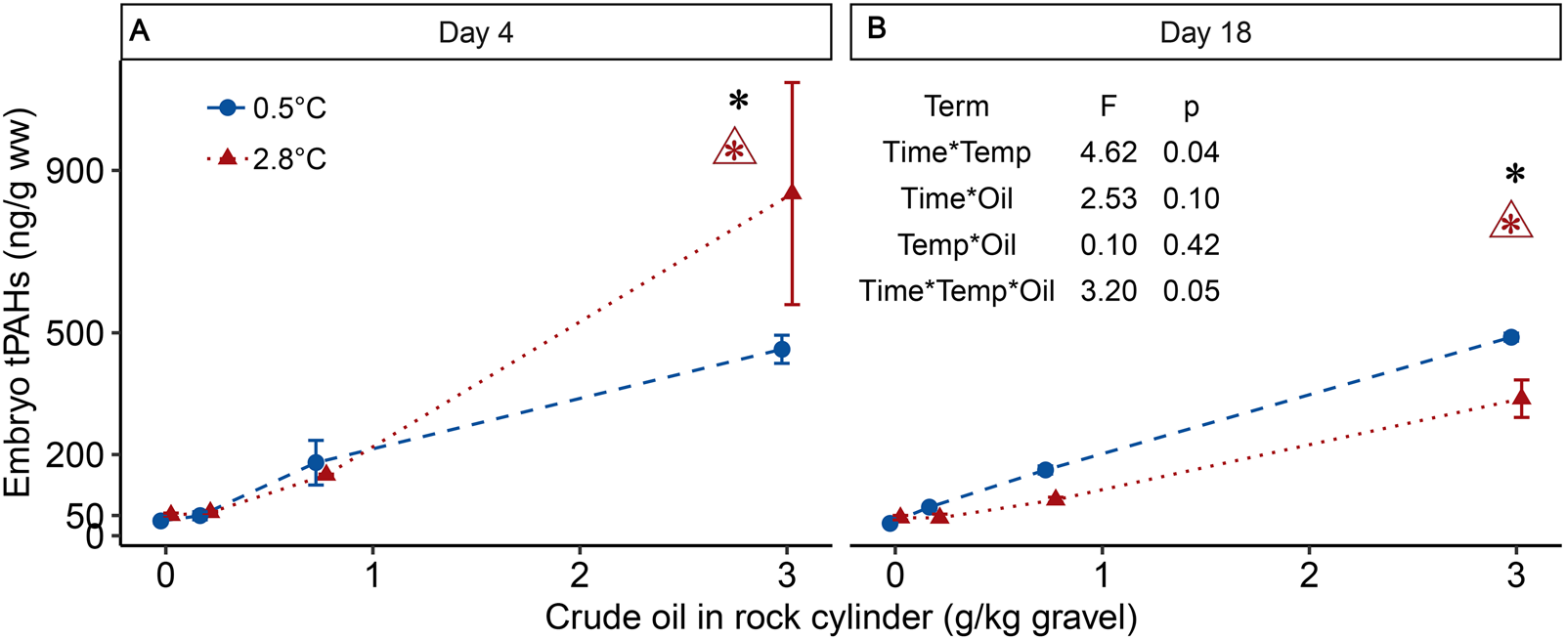
The effect of temperature and WSF crude oil exposure on tPAH levels (ng/g ww) in embryos on (**A**) day 4 and (**B**) day 18 displayed as treatment means (± SEM depicted as bars, each treatment is represented by two independent pools of two incubators), plotted on a log scale. Colors, symbols, and line types distinguish the temperature groups. The best linear mixed effect (LME) model was tPAH ~ time*factor(temperature)*factor(oil treatment) and ANOVA f-values and p-values for the interactions are displayed. An asterisk (*) above a treatment group indicates a statistically significant difference from the unexposed group, a triangle (open triangle) with an asterisk indicates a statistically significant interaction between temperature and crude oil exposure at the adjacent crude oil treatment group. Embryos were at different development stages during the sampling; Day 4: late cleavage stage (0.5 °C) and blastulation (2.8 °C); Day 18: early organogenesis (0.5 °C) and mid organogenesis (2.8 °C).

Accumulation of tPAHs in embryos was elevated in the warm, high oil treatment (Fig. 2, Supplementary Table S2ab). The highest levels of tPAHs in embryos were measured in the warm, high oil treatment [843.0 ± 193.45 ng/g wet weight (ww) (mean ± SEM)] at day 4 (Fig. 2A); accumulated tPAH levels in this treatment decreased by 60% over 2 weeks (337.7 ± 32.7 ng/g ww tPAHs at day 18; Fig. 2B). Embryos in the cold, high oil treatment accumulated 459.4 ± 24.5 ng/g ww tPAHs by day 4; levels were elevated by 6% on day 18 (489.3 ± 6.9 ng/g ww).

### Effects on embryos

During the period of fertilization through gastrulation, no difference in mortality was seen in temperature groups or crude oil treatments with mean instantaneous mortality rates in the range of 0.013–0.020 day^-1^ (Supplementary Fig. S2). During organogenesis, the mortality rates decreased for all treatments (0.0008–0.0057 day^−1^; Supplementary Fig. S2); however, exposure to high oil and warmer water temperature negatively affected survival in embryos.

**Figure 3:**
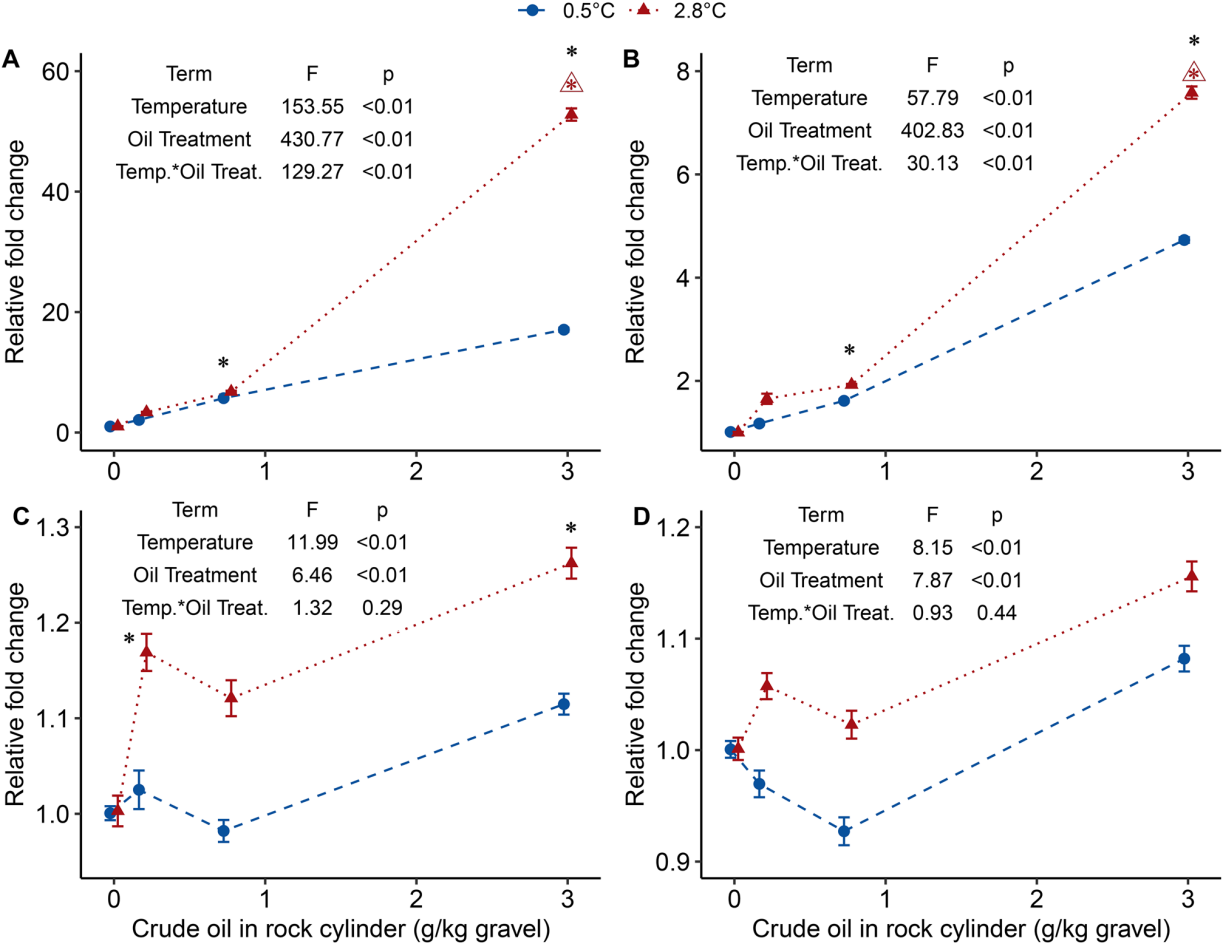
The effect of temperature and WSF crude oil exposure on gene expression of (**A**) *cyp1a*, (**B**) *cyp1c1*, (**C**) *hsp 70*, and (**D**) *hsp 8* measured by qRT PCR in embryos just prior to hatch (day 28 at 2.8 °C and day 48 at 0.5 °C). Data is displayed as treatment means (± SEM depicted as bars, each treatment is represented by four independent incubator pools of 50 embryos each). Colors and symbols indicate temperature groups and dashed and dotted lines represent the LME model trend line for each temperature group. The terms of the linear mixed effect model for each gene and the ANOVA F-values and p-values are displayed in each panel. An asterisk (*) above a treatment group indicates a statistically significant difference from the unexposed group, a triangle (open triangle) with an asterisk indicates a statistically significant interaction between temperature and crude oil exposure at the adjacent crude oil treatment group.

Synergistic effects on biotransformation related genes were measured in the warm, high oil treatment with enhanced expression of gene transcripts for *cyp1a* (Fig. 3A; maximum relative fold change of 52) and *cyp1c1* (Fig. 3B; maximum relative fold change at 7.6)*.* The effects of oil exposure and temperature were measured for *hsp 70* (Fig. 3C) and *hsp 8* (Fig. 3D) with a maximum relative fold change of 1.3 and 1.2, respectively.

**Figure 4:**
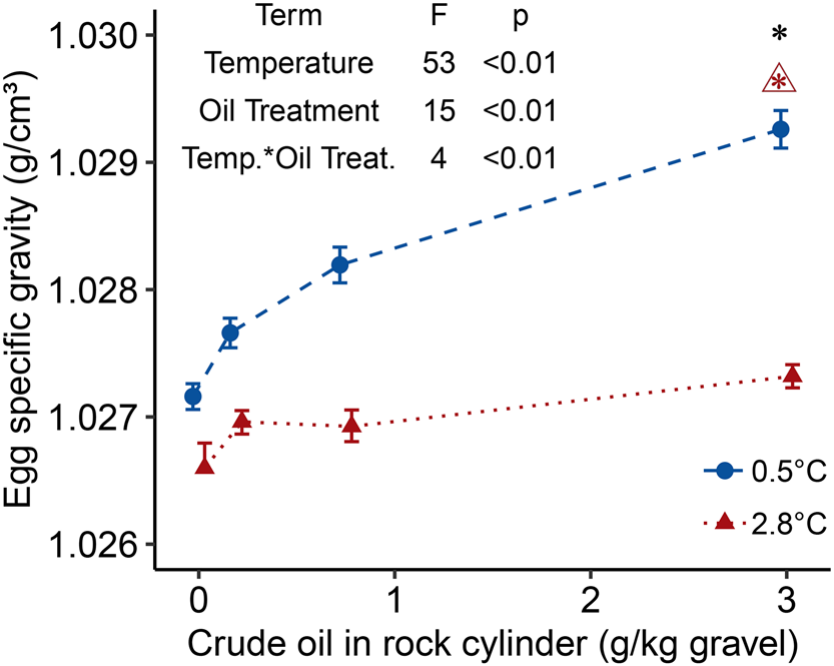
The effect of temperature and WSF crude oil exposure on embryo specific gravity (g/cm^3^) before 50% hatch in the respective temperature groups (day 29 in 2.8 °C and day 56 in 0.5 °C) displayed as treatment means (± SEM depicted as bars, each point includes mean values from four incubators, each with 20 embryos). Colors and shapes distinguish temperature groups. Fitted dashed lines are the results of a GLS model using incubator as a random factor. The terms of the best gls model for embryo specific gravity and the ANOVA test F-values and p-values are displayed. An asterisk (*) above a treatment group indicates a statistically significant difference from the unexposed group, a triangle (open triangle) with an asterisk indicates a statistically significant interaction between temperature and crude oil exposure at the adjacent crude oil treatment group.

Exposure to crude oil resulted in an increased specific gravity of embryos by as much as 0.002 g/cm^3^ in the cold, high oil treatment (97% faster sinking speed in the dilute seawater medium) compared to the cold control group (Fig. 4). In the warm group, the high oil treatment embryos sank 14% faster (0.001 g/cm^3^ difference) than control embryos.

### Effects on yolk sac larvae

**Figure 5:**
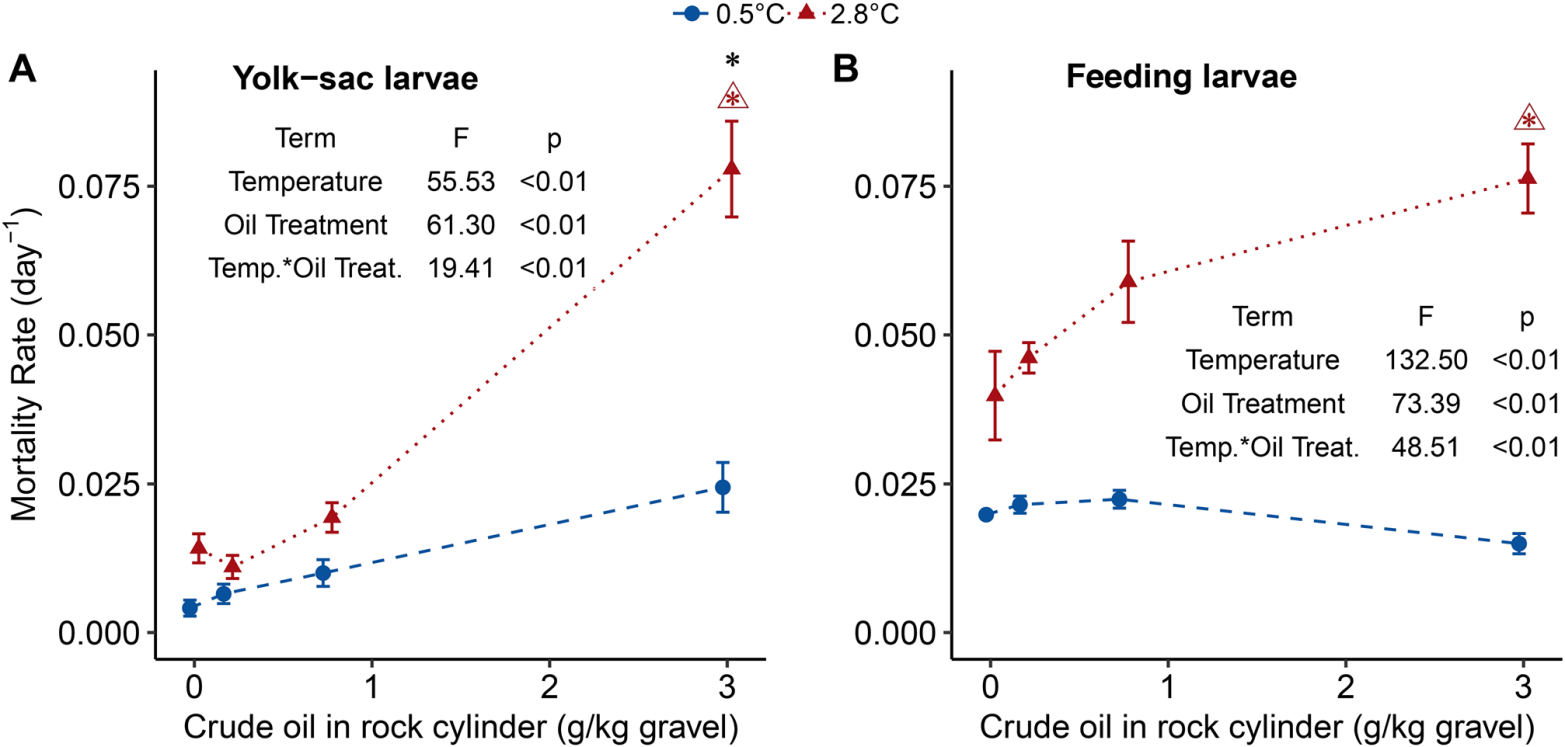
The effect of temperature and WSF crude oil exposure on instantaneous mortality rates for (**A**) yolk-sac larvae [day 27–46 (2.8 °C); day 40–68 (0.5 °C)] and (**B**) feeding larvae [day 47–52 (2.8 °C); day 69–76 (0.5 °C)]. Data is displayed as treatment means (± SEM depicted as bars, each treatment is represented by four independent incubators). Colors and symbols indicate temperature groups and dashed and dotted lines represent the trend line for each temperature group. The terms of the best LME models for each stage are displayed in the respective panels with the associated ANOVA test F-values and p-values. An asterisk (*) above a treatment group indicates a statistically significant difference from the unexposed group, a triangle (open triangle) with an asterisk indicates a statistically significant interaction between temperature and crude oil exposure at the adjacent crude oil treatment group.

**Figure 6:**
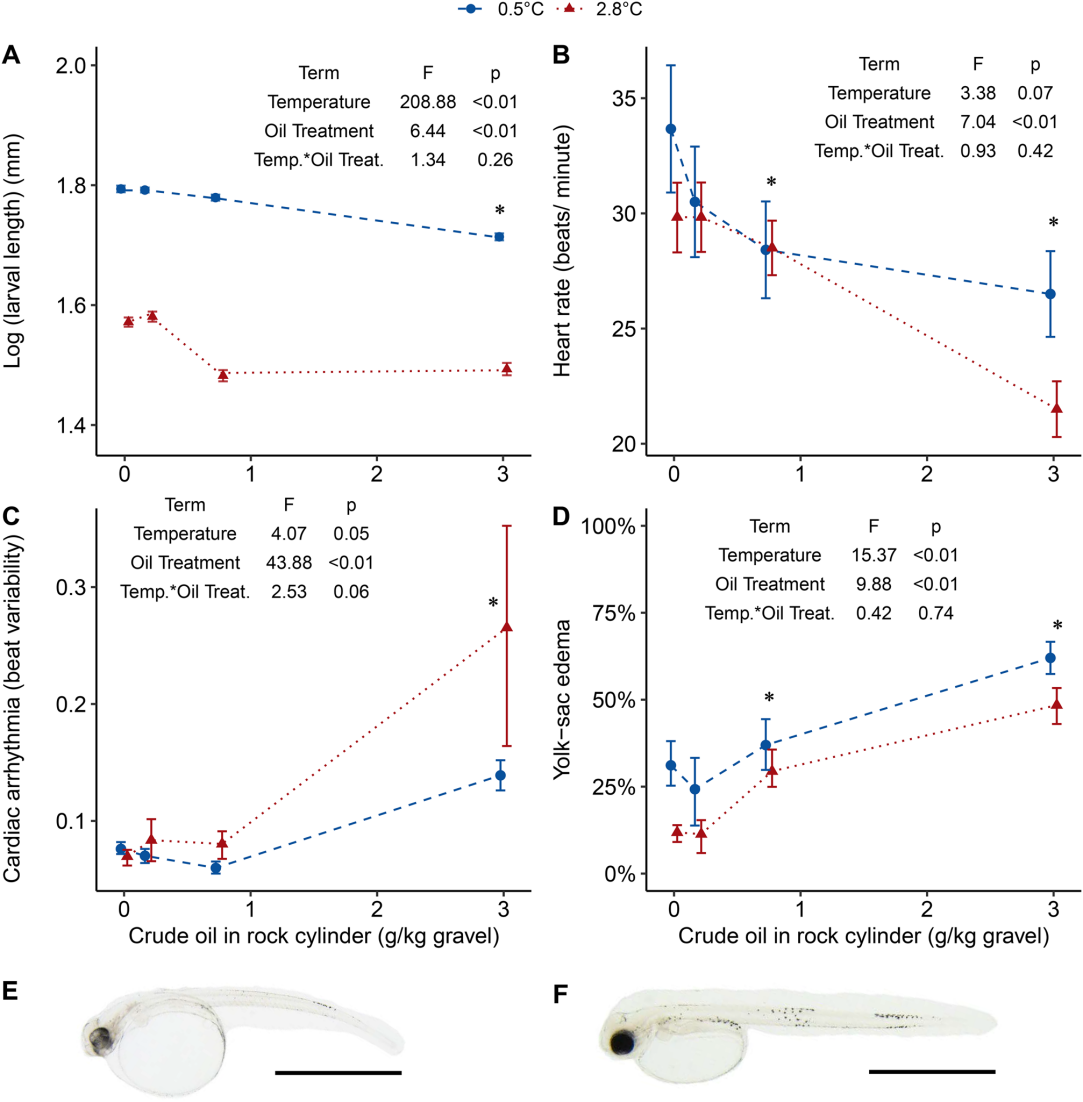
The effects of temperature and WSF crude oil exposure on yolk sac larvae: (**A**) log (length in mm) at day 28 for 2.8 °C and day 50 for 0.5 °C; (**B**) heart rate and (**C**) cardiac arrhythmia at day 29–30 for 2.8 °C and Day 47–48 for 0.5 °C displayed as interbeat variability; (**D**) the prevalence of yolk sac edema; (**E**) a warm-reared unexposed larvae; and (**F**) a cold-reared unexposed larvae at day 28 for 2.8 °C and day 50 for 0.5 °C with a scale bar representing 2 mm. Fitted dashed lines are the results of gls models using incubator as a random factor for panel (**A**–**C**). For all panels, colors, line types, and shapes distinguish temperature groups and treatment means are plotted [± SEM depicted as bars, each point includes four incubator means calculated from 30 larvae (panel **A**) or three larvae each (panel **B** and **C**)] per incubator. Panel (**D**) presents treatment means (± SEM depicted as bars, each point includes deformity scores (*i.e.,* percentage of larvae afflicted) from four incubators) and the fitted line is a result of the LME model using incubator as a random factor. The terms of the GLS model for larval length, heart rate and arrhythmia and the lme model for prevalence of yolk-sac edema are displayed in the respective panels with the ANOVA test F-values and p-values. An asterisk (*) above a treatment group indicates a statistically significant difference from the unexposed group. In panel (**E**) and (**F**), the scale bar is 2 mm.

The warm-incubated embryos hatched earlier and over a shorter duration (day 27–32) compared to the cold group (day 43–65; Fig. S3C) with no observed effect of crude oil exposure on timing of hatch. In the yolk sac stage, warm temperature and high crude oil exposure acted synergistically on larval mortality (Fig. 5A). Adverse effects of oil treatment and temperature were observed in yolk sac larval length with no interaction (Fig. 6A). On average, the warm, control yolk sac larvae had 60% greater yolk sac area (Supplementary Fig. S4) and were 20% shorter relative to the cold, control yolk sac larvae (Fig. 6A). Embryos in the cold, high oil treatment hatched at a reduced length (5.55 ± 0.03 mm) compared to the control larvae of the same temperature (6.01 ± 0.03 mm), but no such trend was seen in the warm treatment (4.81 ± 0.04 mm and 4.45 ± 0.05 mm for control and high treatment, respectively).

Heart rate in newly hatched larvae decreased with increasing exposure to crude oil (Fig. 6B). In the high oil treatments, heart rate averaged 28% and 18% lower than in the warm and cold control larvae, respectively. Cardiac arrhythmia increased in warm reared larvae and those exposed to the highest oil treatment (Fig. 6C). Higher incidences of yolk sac edema were seen in high oil treatment larvae in both temperature groups with up to 62% of the yolk sac larvae afflicted in the cold, high oil exposed treatment (Fig. 6D). Furthermore, a higher prevalence of yolk sac edema was exhibited in the unexposed cold-reared larvae compared to the unexposed warm group (Fig. 6D). Temperature affected the development of embryos in a nonlinear fashion. The warm-reared group developed more quickly in the later phases of embryogenesis (i.e., organogenesis), hatching with less-developed jaws, eyes, and hindguts and larger yolk sacs compared to the cold group (Fig. 6E,F; Supplementary Fig. S3).

### Effects on feeding larvae

**Figure 7:**
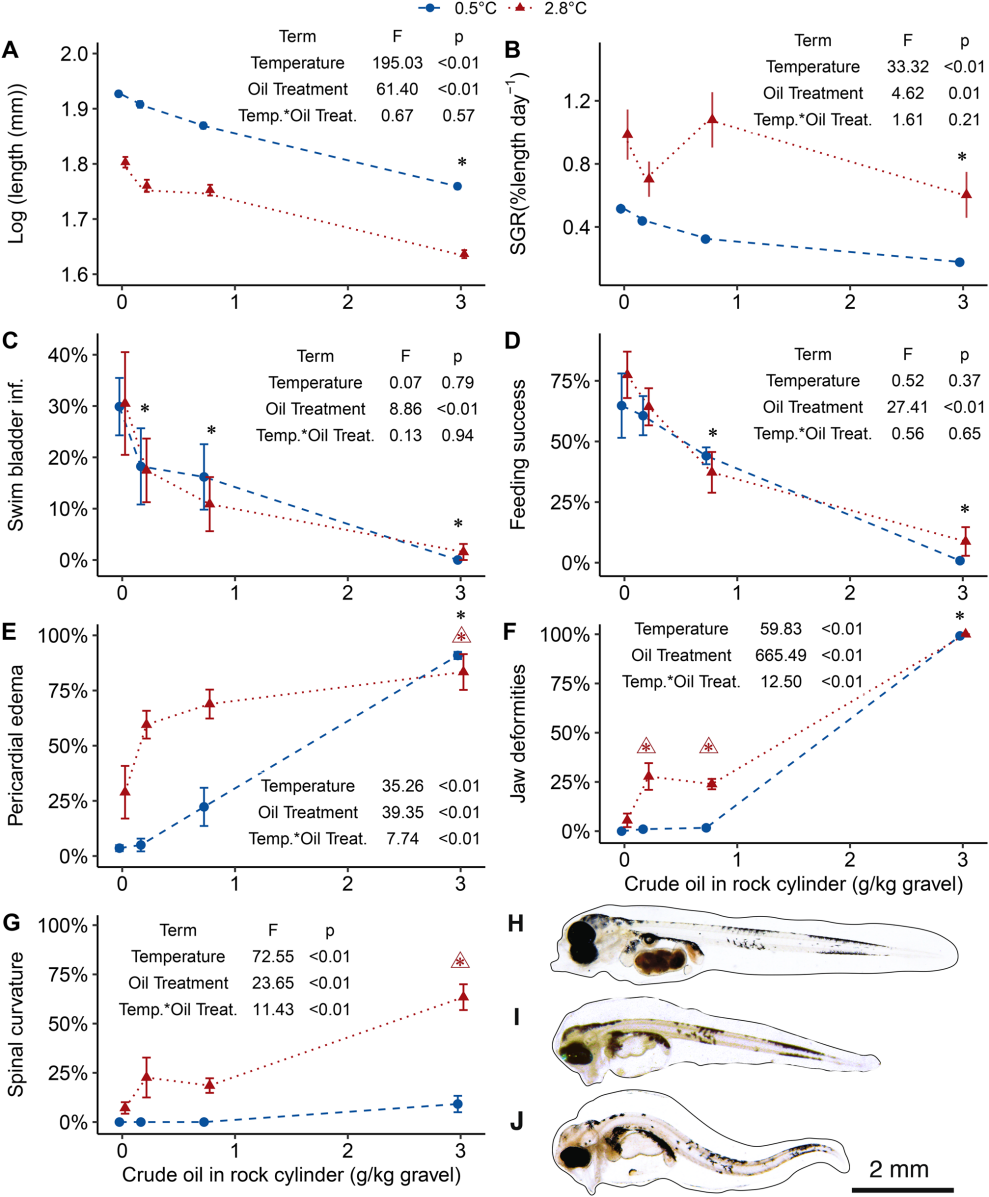
The effects of temperature and WSF crude oil exposure on exogenously feeding larvae (day 52 for 2.8 °C and day 76 for 0.5 °C) morphometrics, development and phenotype: (**A**) log [length (mm)] of larvae and (**B**) Specific growth rate (% length day^-1^) and displayed as treatment means (± SEM depicted as bars, each point includes four incubator means from 30 larvae). Fitted dashed lines are the result of a gls model using incubator as a random factor. The terms of the GLS model for larval length and SGR are displayed in the respective panels with the ANOVA test F-values and p-values. Specific growth rate was calculated between day 28–52 for the warm group and day 50–76 for the cold group. The frequency of occurrence/prevalence of (**C**) swim bladder inflation; (**D**) feeding success; (**E**) pericardial edema; (**F**) jaw deformities; and (**G**) spinal curvature represented as treatment mean percentages (± SEM depicted as bars, each point includes 4 incubator deformity scores). Fitted dashed lines are the results of LME model using incubator as a random factor. The terms of the LME models for each parameters is displayed in the respective panels with the associated ANOVA test F-values and p-values. For all panels, colors, shapes, and line types distinguish temperature groups. An asterisk (*) above a treatment group indicates a statistically significant difference from the unexposed group, a triangle (open triangle) with an asterisk indicates a statistically significant interaction between temperature and crude oil exposure at the adjacent crude oil treatment group. Typical phenotypes of exogenously feeding larva exposed to (**H**) Control oil treatment in the cold group; (**I**) Cold, high oil treatment; and (**J**) warm, high oil treatment.

As larvae began exogenous feeding, synergistic effects of increased temperature and crude oil exposure were evident in mortality rates (Fig. 5B) and spinal and jaw deformities (Fig. 7F,G). The highest survival of first feeding larvae was in the cold, unexposed treatment (55 ± 0.03%) and the lowest survival was recorded in the warm, high treatment (8 ± 0.02%) (Fig. S5). The length of first feeding larvae decreased with exposure to the medium and high oil treatments and exposure to the warm temperature (Fig. 7A).

The specific growth rate, calculated from measurements taken in the yolk sac and first feeding larval stages, was negatively affected by high oil exposure and positively affected by temperature. In the warm group, the highest growth rate was seen in the medium oil treatment (1.07 ± 0.17% of length day^−1^) and the lowest rates were seen in the low and high oil treatments (0.6–0.7%) although not statistically different from the control growth rate (Fig. 7B). Growth rates in the unexposed fish were 92% higher in the warm treatment (0.99 ± 0.16%) compared to cold treatment (0.52 ± 0.03%) using group means. Swim bladder inflation (Fig. 7C) and feeding success (Fig. 7D) were negatively affected by crude oil exposure at the high and medium treatments but were similar across temperature groups.

**Figure 8:**
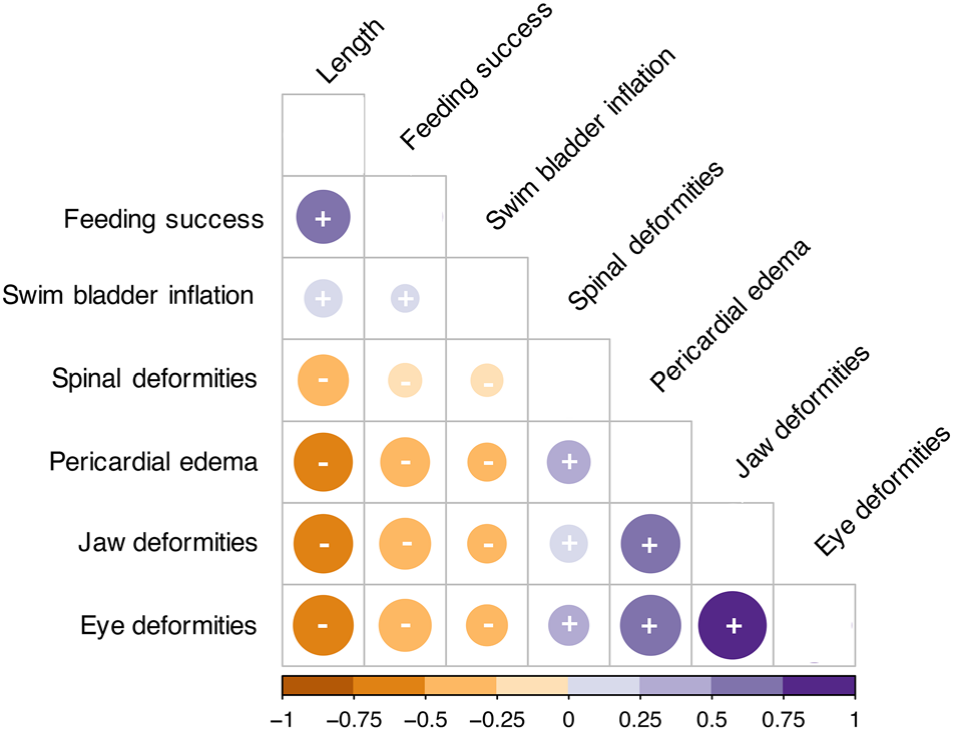
Correlation matrix calculated with the non-parametric Spearman method for biomorphometrics and deformity responses in individual exogenously feeding larvae from all treatments (n = 809 larvae, sampled at day 52 for 2.8 °C and day 76 for 0.5 °C). Dot size indicates strength of correlation coefficient with larger dots equating to higher correlation coefficients. Color and symbols indicate direction of the correlation with positive correlations in purple ( +) and negative correlations in orange (-). P-values are < 0.05 for all correlations.

Synergistic effects of increased temperature and crude oil exposure were observed in the percentage of feeding larvae with jaw and spinal deformities and pericardial edema (Fig. 7E–G). An antagonistic effect of the combined stressors was observed in the prevalence of pericardial edema (Fig. 7E). Larvae from the warm group exhibited a higher incidence of all deformities at lower crude oil concentrations than larvae in the cold-reared group (Fig. 7H–J). Pericardial edema (Fig. 7E), as well as eye (Supplementary Fig. S6) and jaw deformities (Fig. 7F), were present in nearly 100% of larvae exposed to high oil treatment regardless of temperature. In contrast, the medium, warm-reared larvae had 20% or higher incidence of all deformities including spinal deformities compared to the cold group at the same crude oil treatment. Endpoints investigated in feeding larvae were highly correlated (Fig. 8). While effects of temperature and crude oil exposure have been reported in detail, this analysis reveals that physiological endpoints such as length, feeding success, and swim bladder inflation are negatively correlated with the presence of deformities. Jaw and eye deformities are highly positively correlated (correlation coefficient > 0.75), while spinal deformities have the weakest correlations with the other deformities (correlation coefficients range from 0.25 to 0.5).

## Discussion

Exposure to low levels of the WSF of crude oil during embryonic development led to sublethal and lethal effects in polar cod embryos and larvae, and these effects were further amplified by an increase of 2.3 °C in water temperature. Interaction effects of temperature and crude oil exposure were observed in 64% (14 of 22) of the responses measured and spread across all developmental stages from embryos to feeding larvae and at multiple levels of biological organization from gene expression to rates of deformities and mortality (Supplementary Table S6). Within the interaction effects, 43% (6 of 14) of these response interactions found to be strong synergistic effects, most notably in the survival and prevalence of deformities in yolk sac and feeding larval stages. Synergism is hypothesized to be the most likely outcome of simultaneous exposure to multiple stressors, as the increased stressor intensity will likely overcome compensatory mechanisms in the organisms^50^. Multiple stressors, in the case of synergism, have a larger effect on the organism or population than the sum of the individual stressors (i.e., an additive response) and contribute to a better understanding of the biological responses of organisms in a more complex environment^4^.

Global environmental change is not limited to increased water temperature and acute pollution events, as this study presents. Reductions in sea ice extent, ocean acidification, increasing freshwater input, southern species moving northward to alter community structures and other pollution issues like microplastic all stand to affect polar cod^17,51–53^ with possible cascading effects to the entire Arctic marine ecosystem. The reductionist approach to the present work explored the resilience of this species at vulnerable life stages. Studies on juvenile and mature polar cod report higher thermal preferences and tolerance than early life stages^54,55^ and robust physiological response to chronic dietary exposure to crude oil and acute exposure to oil spill response measures^56,57^.

Exposure concentrations of tPAHs in the present study (5–237 ng/L) are within the lower range of tPAHs measured in waters in the years following Exxon Valdez Oil Spill in Prince William Sound, Alaska (129–126,635 ng/L)^58^ and within the range of concentrations measured months after Deepwater Horizon Oil Spill (mean value < 100,000 ng/L)^59^. Caution in interpretation of cause/effect relations should be exhibited as this study has only measured and compared the concentration of 44 PAHs, which make up a small percentage (1.5–3%, Berenshtein et al*.* 2020) of the total hydrocarbons of the WSF of crude oil. tPAH levels explain only 20% of the variation in total petroleum hydrocarbon concentration from 29 water samples collected following the Deepwater Horizon Oil Spill^60^. Given this weak relationship and the low tPAH levels measured in this study, it is likely that other compounds in crude oil are causing toxicity.

The reduction in heart rate, increased heart arrhythmia, and increased incidence of pericardial edema are well-described cardiac impairments resulting from exposure to petroleum in fish ELSs^22,61^. Observed deformities of the spine, eye, and jaw could be linked to reduced cardiac function^62^; however, other mechanisms have been suggested, such as impaired neural crest development^63^. Reduced cardiac function is linked to reduced cardiorespiratory performance, persisting even in adult and juvenile fish exposed to crude oil as embryos^42,64^. Furthermore, reduced survival and growth of pink salmon (*Oncorhynchus gorbuscha*) released into the marine system after exposure to the WSF of crude oil as embryos has been reported, providing substantial evidence for the delayed effects of embryonic exposure to crude oil^65^.

In the water of the warm, high oil treatment bulk oil was present, likely due to the temperature effects on the relative viscosity or pour point and thus retention of the fresh Kobbe crude oil in the gravel cylinders^66,67^. This same phenomenon was not observed in the cold, high oil treatment and so we proceed cautiously with our analysis of potential synergistic effects, as the exposure compound profile differed between temperatures. As evidenced by the two-dimensional gas chromatography analysis, we see that using standard PAH quantification techniques in a whole crude oil exposure scenario risks underestimating the contribution and the mechanistic understanding of the effects of semi-polar, polar, and monoaromatic compounds, which have been demonstrated to be of toxicological significance^68^ and worthy of additional investigation^69^. The presence of oil droplets in the system alone may not be linked to increased toxicity as ELS studies have shown embryos to be mainly affected by the WSF of crude oil^70,71^; however, oil droplets have been observed to contribute to acute and sub-lethal effects in Atlantic cod ELS^72^. Crude oil droplets, when present, adhere to the chorion of polar cod embryos^37^ and may increase the internal concentrations of PAHs, especially larger and alkylated homologues^70^.

The upregulation of genes encoding for *cyp1a* and *cyp1c1* indicates that crude oil-derived compounds were biologically available^43^ and that biotransformation mechanisms are in place early in polar cod embryogenesis, as seen in Atlantic cod embryos^73^. The warm, high oil-treated embryos exhibited a robust up-regulation of biotransformation genes *cyp1a* and *cyp1c1* prior to hatch (day 28), evidence for metabolism of PAHs and an explanation for the decreased tPAH accumulation in embryos from day 4 to day 18. Alternatively, this could be explained by the declining PAH concentrations in the water. However, this is an interpolation between the two sampling points, and further work is needed to confirm a cause-effect relationship. While synergistic effects of warm water and high oil treatment were seen in molecular responses in the late embryo stage, there were no interactions observed between the effects of temperature and crude oil exposure on early embryonic survival.

The adverse effect of crude oil exposure resulting in higher specific gravity of embryos, measured as embryo sinking speed in less saline water, may have implications on early life survival and horizontal and vertical dispersal of ELSs in the ocean and under the ice^74^. Polar cod embryos may maintain low specific gravities to provide a suitable position near sea ice and in the low density, Arctic surface waters^75^. Reduced buoyancy was also observed in Mahi-mahi embryos exposed to crude oil and higher temperatures and was suggested to be adaptive as embryos actively avoid unsuitable conditions at the surface, likely through rapid ion exchange^76^. While a mechanistic understanding of changes in embryo buoyancy is outside the scope of this study, exposure to crude oil has likely disrupted osmoregulation of the normally hyperosmotic, buoyant embryos. In haddock, embryonic exposure to crude oil caused differential expression of osmoregulation gene pathways^45^. While depletion of yolk reserves or altered lipid metabolism would likely alter embryo density^74^, yolk sac area measurements in larvae measured shortly after hatch did not statistically differ with crude oil exposure. Differences between the temperature groups may be explained by changes in body structure (i.e., greater length at hatch and higher specific gravities in the cold reared group) as proteins are the heaviest component of embryos^74^. Notably, in the warm treatment, the magnitude of the effect of crude oil exposure on embryo buoyancy was reduced and may suggest a protective effect of increased temperature. It is not likely that selection through mortality was strong as treatment-dependent mortality was first observed in late embryogenesis.

Lower embryonic survival and premature hatching of the warm reared larvae, determined by the smaller size, undeveloped jaws, lightly pigmented eyes, and simple digestive system compared to the cold-reared larvae, corroborates the high thermal sensitivity of polar cod^28^. Development times reported in this study agree with other polar cod experiments and observations around the Arctic^40,75^. Maximum polar cod embryo survival occurs between 0 and 1.5 °C with a reduction in embryo survival seen at 3.0–3.5 °C^17,28^, confirming that the study presents data from the higher end of the thermal window for polar cod embryos at 2.8 °C. Premature hatching with increased temperature is a phenomenon also seen in polar cod in Alaskan waters and Atlantic cod^28,78^. The larger size and more advanced developmental stage in the cold-reared larvae resulted in exogenous feeding shortly after hatch. Increased prevalence of yolk sac edema in the unexposed, cold reared larvae compared to the warm reared larvae was inconsistent with other morphological responses in this study and may be related to temperature specific developmental patterns. The heat shock response genes (*hsp 70* and *hsp 8*) up-regulated in the warm treated embryos may indicate a greater stress response. Hatching at smaller sizes may leave warm-reared larvae more vulnerable to predation and less fit-to-forage in the wild^79^. However, larval growth rates were higher in the warm-reared larvae and the prevalence of swim bladder inflation and food present in the stomach of larvae did not differ between the temperature groups. The difference in the timing of developmental events (i.e., heterokairy in hatching, 1^st^ feeding, or reaching a greater size) is evidence of developmental plasticity with increased temperature^80^ and these changes may prove advantageous in the Arctic ecosystem under climate change, such as the ability of larvae to forage on earlier spring blooms in production^81^.

In fact, we observed higher larval growth rates in warmer temperatures, which potentially place surviving, more tolerant individuals on better survival trajectories after hatch. Growth and survival rates are both stage-specific and temperature-dependent, even within the larval stage of polar cod^82^. Accelerated larval growth is likely associated with higher energetic demands and may increase the vulnerability of growing larvae to alterations in food quality and availability^28,83^, a predicted change in the Arctic zooplankton community composition with ongoing warming^84,85^. Field studies consistently report temperature as the main driver of polar cod ELS abundance, with fewer fish found in warmer waters^35,81,86^. Yet, field observations suggest a wider thermal tolerance (− 1.7–6.5 °C) for spawning areas and larvae occurrence^30^ than lab studies support^17,28,77^.

Subtle stress levels are hypothesized to lead to more antagonist effects while synergism is more common at high-stress levels, as organisms are able to compensate^87^. Our experimental results revealed interaction effects to be synergistic in 6 of 14 responses. Statistically significant antagonist interaction between the two stressor types at the high oil treatment level was observed in two responses—embryo specific gravity and in the prevalence of pericardial edema response in feeding larvae. At lower crude oil exposure levels, responses were synergistic for pericardial edema indicating that a potential maximum of larvae with this phenotype was reached at 83%. After elevated mortality during the yolk sac and feeding larval periods of the warm, high oil group, it is possible that the remaining larvae were more robust to the multiple stressor scenario^50^. Even with higher rates of deformities (i.e., of the jaw, eye, and spine) at lower oil exposures, warm treated fish were as able to feed and reached similar developmental milestones, such as swim bladder inflation, as cold reared larvae exposed to oil. Higher mortality rates in the warm treatment leave the more robust, albeit skeletally deformed larvae in these treatments. The high incidence of craniofacial deformities for polar cod exposed to low concentrations of crude oil has been observed in other studies^36,37^. Deformities observed likely hamper the swimming and foraging ability in larvae resulting in the high correlation between feeding success and presence of deformities. The timing of the loss of severely deformed larvae matches temperature-corrected starvation windows determined experimentally for polar cod larvae (35–50 days post hatch)^28^. Investigation in the chronic physiological effects of subtle multi-stressor exposure on growth, energy use, and behavior would further this research and aid in addressing the possible long-term toxic responses and population-level effects.

A 2.3 °C increase in temperature combined with a crude oil exposure resulted in synergistic effects, especially on larval survival and on the frequency of deformities. The warm, high oil treatment likely altered the exposure from the water soluble fraction to one containing bulk oil microdroplets detected only through two-dimensional gas chromatography. Crude oil exposed embryos exhibited increased specific gravity, and larvae were afflicted with deformities of the jaws, eyes, and spine, and a reduced heart rate. Despite the expectation that increases in temperature within the polar cod embryonic thermal window and low levels of crude oil exposure would result in moderate responses^39,88^, the magnitude of the effects observed in this study is large, further supporting the vulnerability of polar cod ELS to environmental stressors such as increased temperature and petroleum pollution. Under a multi-stressor scenario, polar cod will likely be at a disadvantage in the changing Arctic environment and interactive effects will challenge predictive models and the ability to protect sensitive species and ecosystems.

## Methods

The collection of wild adult polar cod was carried out with approval from the Norwegian Fisheries Department (17/14068). Experimentation was performed in accordance with guidelines and regulations approved by the Norwegian Food Safety Authority (17/243813) and reporting herein follows the recommendations in the ARRIVE guidelines^89^.

### Broodstock collection and husbandry

Mature polar cod were captured south of Northeast Land, Svalbard (78°55′N, 23°40′E) using a bottom trawl fitted with a live fish box^90^ aboard the RV *Helmer Hanssen* in November of 2017. Fish were kept on deck in four 400L flow-through seawater tanks during the 8-day transport to the Aquaculture Research Station in Tromsø, Norway. Broodfish were acclimated to the laboratory in a 4000L tank under a daylight cycle of 80°N at 2.3–2.5 °C, fed to satiation with frozen wild *Calanus* zooplankton (Calanus AS) and treated with Halamid disinfectant solution (0.01 g/L) as needed. Experimental timing was based on the gravid status of adults, as determined from daily pilot strip spawns of anesthetized females.

### Oil exposure preparations and chemical analysis

The WSF of crude oil was formed through the contact of seawater with oiled gravel^43,64^ to simulate an oil spill leeching from the melting sea ice^36^. Before oiling, gravel was cleaned by first sieving (4–8 mm diameter), soaking in 1 M HCl for 2 h, thorough rinsing with clean water, overnight immersion in 90% ethanol, a second thorough rinsing with clean water, and then dried at 60 °C. Fresh Kobbe crude oil from the Goliat field, Barents Sea (provided by SINTEF Ocean) was distributed at concentrations of 0, 0.19, 0.75, and 3 g crude oil/ kg gravel (corresponding to control, low, medium and high treatments, respectively) and set to dry at 0 °C for 72 h. Polyvinyl chloride (PVC) cylinders (n = 8, 1 m tall) were loaded with 11 kg of oiled gravel (or clean control gravel) with two cylinders for each oiled gravel concentration. Cylinders were further divided into two temperature groups, such that each temperature group contained one of each of the four oiled gravel concentrations. Cylinders were capped with aquarium filter floss to catch any oil droplets and sealed to prevent overflow. Seawater at 34‰ was filtered at 20 µm, UV cleaned, cooled to either 0.5 ± 0.4 °C or 2.8 ± 0.3 °C (now referred to as “cold” and “warm” treatments, respectively), and stored two 300 L header tanks at to ensure stable temperatures. The cooled seawater was flushed from header tanks up through the gravel at 100 L/h for 76 h to allow for the loss of the most water–soluble oil compounds such as the acutely toxic BTEX (benzene, toluene, ethylbenzene, and xylene) compounds. Cylinders were prepared in advance of the experiment start with one cylinder per oil (n = 4)/ temperature combination (n = 2, 8 columns in total) and were frozen at -20 °C after initial flushing. Immediately before the start of exposure, cylinders were thawed, and outflow from each cylinder was distributed into four replicate conical incubators (25 L capacity; flow at 25 L/h, constant aeration) (Supplementary Fig. S7). The experiment included 32 experimental incubators. Incubators were randomly spatially distributed with regard to crude oil exposure within the two temperature treatments. Temperature (Supplementary Fig. S3) and light levels were logged every minute in two randomly-selected incubators within each temperature group, and oxygen was monitored bi-weekly to confirm > 98% of saturation. The light cycle was adjusted weekly to that of 80°N.

### Experimental setup

In January 2018, ripe polar cod were taken from the laboratory acclimated broodstock, sedated (MS 222, 50 mg/L), and strip spawned. Visually viable eggs from 49 females and milt from 21 males were collected. Milt samples were pooled in equal ratios. Eggs from each female were divided into two groups of the same volume and pooled in equal ratios. Fertilization of each egg group (450 mL) was conducted within a temperature-controlled water bath (either 0.5 °C or 2.8 °C) by adding 10 mL of the milt pool and 3 L of seawater. After 10 min, egg groups were rinsed of milt and distributed across 16 experimental incubators (28 mL/incubator) corresponding to the fertilization temperature. Each incubator held 8000–10,000 eggs at the start of the experiment. Embryos were followed for 170 days post fertilization; however, this work presents endpoint measurements from fertilization to first feeding within each temperature group (day 52 at 2.8 °C and day 76 at 0.5 °C).

The developmental progress of embryos was tracked daily by imaging three embryos or larvae from a 5th control incubator, held at each corresponding temperature, which were not included in the main experiment (Supplementary Fig. S7). Embryos were photographed at multiple magnifications using a Leica M205 C stereomicroscope and camera (Leica, MC 170 HD). Sampling events from the 32 experimental incubators were performed at similar developmental stages in the two temperature groups based on the stage determined from embryos and larvae in the additional control incubators. Staging of embryogenesis (cleavage stage, blastulation, gastrulation, and organogenesis) and of the larval stages (yolk sac larvae and preflexion/feeding larvae)^91^ is described in greater detail in Supplementary Table S3. Collection and enumeration of opaque dead or dying embryos or larvae from the bott of all incubators was done daily. Instantaneous mortality rates (M) were calculated for the developmental stages at each temperature group according to the development of the control embryos or larvae from the additional incubators using the equation:

1 $${\text{M}} = \frac{{\ln N_{t} - \ln N_{o} }}{ - t}$$

where N_t_ is the abundance at the end of the developmental period, N_o_ is the abundance at the start of the developmental period, and t is the number of days spent in the developmental period^92^. Abundance was determined using a total tally of dead, sampled, and alive larvae over the entire experiment.

Enriched rotifers (5 ind/mL density in incubators) and green water (Nanochloropsis, Reed Mariculture) were introduced to the incubators when jaw formation was observed in each temperature group (day 35 and day 56 at the warmer and colder treatments, respectively; Supplementary Fig. S3). Larvae were transitioned on to *Artemia* nauplii (2 ind/ml density in incubators) as their intestines began to curl (day 47 and day 60 at the warmer and colder treatments, respectively).

### Analytical chemistry

#### Water collection and polycyclic aromatic hydrocarbon (PAH) extraction

Water samples for chemical analysis were collected directly from the cylinder outflow at the start of the experiment (day 0) and days 4, 10 (warm treatment only), 18, 28 (warm treatment only), and 56 for each temperature and crude oil concentration. Samples were acidified (15% HCl) and stored in the dark at 4 °C until analysis. The organic phase was extracted from the water by first transferring the entire sample to separatory funnels and rinsing sample bottles with dichloromethane (DCM, 60–90 mL depending on sample size). Surrogate internal standards (25.08 ng naphthalene-*d*8, 5.00 ng phenanthrene-*d*10, 4.86 ng chrysene-*d*12 and 5.08 ng perylene-*d*12) were added to each sample. The organic and aqueous phase were allowed to separate after shaking, and the organic phase was transferred to a flask with sodium sulfate. This was repeated two more times before the organic phases were combined and concentrated using a TurboVap evaporator. Before analysis, recovery internal standards (fluorene-*d*10 and acenaphthene-*d*10) were added to samples. A laboratory blank (MilliQ water) was included with each sample set.

#### Embryo collection and PAH extraction

On day 4 and day 18, viable embryos (n = 25) were sampled from each incubator and combined with another incubator of the same treatment such that each unique treatment was represented by two sample pools of 50 embryos. Sample pools were immediately frozen at − 80 °C until analysis. Upon analysis, embryos were weighed within 0.01 mg accuracy and the organic compounds were extracted following a method developed by Sørensen and colleagues^93^. Briefly, surrogate internal standards (same as the water samples) and sodium sulfate were added before the samples were homogenized with *n*-hexane-dichloromethane using a disperser (IKA 10 basic ULTRA-TURRAX), vortexed, and centrifuged. Supernatants containing the organic extracts were collected and cleaned using solid-phase extraction with silica (500 mg, Agilent Bond Elut SI, Agilent Technologies, USA), and eluted with *n*-hexane-dichloromethane (1:9, v/v, 6 mL). Purified extracts were concentrated on a heat block (40 °C) under a gentle N_2_ steam. Prior to analysis, recovery internal standards (same as the water samples) were added to the purified extracts. A laboratory blank (empty sample vial) was included with each sample set.

#### PAH identification and quantification

Samples were analyzed by an Agilent 7890 gas chromatograph (GC) coupled with an Agilent 7010B triple quadrupole mass spectrometer fitted with an EI source (70 eV) and collision cell (Agilent Technologies, Santa Clara, CA, USA). Two GC-columns were coupled in series through a purged ultimate union. Samples (1 µL) were injected at 310 °C splitless and carried by high purity helium at a constant flow (1.2 mL/min). The temperature started at 40 °C and increased in intervals until reaching 330 °C when the first column was back-flushed. N_2_ was used as collision gas and helium was used as a quench gas. Target PAHs were identified by two unique multiple reaction monitoring transitions and quantified by the most intense peak^94^. Alkyl PAH clusters were determined by multiple reaction monitoring using transitions from the molecular ion^94^. Parent PAH compounds were quantified by quadratic regression of a 12-level calibration curve (0.01–250 ng/mL), while alkyl PAH groups were quantified by the response factor calculated for a methyl-substituted PAH reference compound. Standards were run for each of the 12 sample injections. The level of detection is reported in Table S1 for each PAH and ranged from 0.0001 to 1.75 ng/L (mean 0.143 ng/L). Concentrations are described using total PAHs (tPAHs) as clusters of alkylated homologs.

#### Two-dimensional gas chromatograph—mass spectrometry

Water samples from the high and control treatment at day 0, 4, (10 warm only), and at end of embryonic period at both temperatures were analyzed by an Agilent 7890B gas chromatograph coupled with an Agilent 7250 quadrupole time of flight mass spectrometer fitted with an EI source and collision cell (Agilent Technologies, Santa Clara, CA, USA). Samples (1µL) were injected at 250 °C splitless. The carrier gas was high purity helium at constant flow (1 mL/min). First dimension separation was achieved using an Agilent J&W DB-1MS column (30 m × 0.25 mm × 0.25 µm). A Zoex ZX2 cryogenic modulator was used to trap and transfer continuous fractions from the first to the second-dimension columns. The hot jet pulse was constantly + 50 °C offset from the oven temperature with a pulse of 350 ms. Second dimension separation was achieved using an Agilent J&W DB-17MS column (1.5 m × 0.25 mm × 0.25 µm). The oven temperature was kept at 90 °C for 1 min, then ramped to 300 °C by 2 °C/min and held for 5 min. The transfer line temperature was 300 °C and the ion source temperature was 230 °C. The source was operated at 70 eV, scan speed was 50 Hz, and scan range was 50–450 m/z. Data were collected in Agilent Masshunter and processed using GCImage.

### Embryonic endpoints

#### Gene expression

In the early phases of hatching between the developmental stages of 100% tail curl and formation of a ‘golden eye’ (Supplementary Figure S2; day 28 at 2.8 °C and day 48 at 0.5 °C), 100 embryos were sampled out of each incubator and frozen at − 80 °C for gene expression analysis. Embryos were chosen for gene expression analysis to avoid the earliest hatching larvae and for morphological and developmental consistency across temperature groups. Quantitative reverse-transcription PCR was performed for 4 genes (*cyp1a, cyp1c1, hsp8,* and *hsp70*) on RNA extracted from embryos after homogenization in Trizol buffer and the addition of chloroform. RNA was extracted and cleaned using an RNeasy mini kit (Qiagen), with additional DNAase treatment. Between 706 and 4195 ng/µL RNA was extracted from each sample (mean 2868 ng/µL RNA) with 500 ng of RNA used to reverse transcribe to cDNA. Control genes had no significant treatment effects on cycle threshold (Ct) values. Relative fold change was calculated using the efficiency-adjusted ΔΔCt method^95^ from 3 control genes (*b-actin, elf1a,* and *rpl4*) and geometric means of relative fold changes from all control genes are reported.

#### Embryo specific gravity

Twenty embryos were randomly sampled from each incubator prior to 50% hatch in both temperature groups (day 29 at 2.8 °C and day 56 at 0.5 °C). Embryos were transferred with a minimum amount of water to a 100 mL glass graduated cylinder filled with diluted seawater (28‰) at either 0.5 °C or 2.8 °C and the time it took for the embryo to sink 5.5 cm was recorded. The 28‰ seawater was found to be appropriate for observing sinking speeds of the positively buoyant embryos reared at 34‰. The time and distance that embryos descended in the water column were converted into a sinking speed (cm/s). Further conversion of sinking speed to embryo specific gravity was performed using Stoke's Law of fluid dynamics in the following equation:

2 $${\text{Embryo specific gravity}} \left( {\frac{{\text{g}}}{{{\text{cm}}^{3} }}} \right) = \frac{{18 \upmu *{\text{sinking speed}}\left( {\frac{{{\text{cm}}}}{{\text{s}}}} \right)}}{{gd^{2} }} + \uprho {\text{w}}$$

where the kinetic velocity of seawater (µ) at 28‰ was extracted from Table 25 in Riley and Skirrow^96^ based on temperature, g is the acceleration due to gravity, an average embryo diameter (d) of 1.63 mm measured from a sub-sample of embryos, and ρw is the density of seawater^97^.

### Sampling of yolk sac larvae

Embryos and larvae were collected using a 1 m long, 4 cm diameter open plastic tube inserted vertically into each aerated incubator. The top of the tube was sealed and a 250 mL sample was collected daily to determine the proportion of larvae to embryos during the period where both stages were observed simultaneously in the incubators. On days 28 and 50 in the warm and cold groups incubators, respectively, the sampling by tube method was repeated until 30 larvae were collected from each incubator. Larvae were subsequently sedated and photographed at 1.6× magnification. Total length (to the nearest 0.01 mm), yolk sac area (freeform traced from picture, in mm^2^), and presence of yolk sac edema (Table S4) were assessed from pictures for each individual larva using ImageJ^98^.

#### Heart rate and arrhythmia

Newly hatched yolk sac larvae were sampled from each incubator at 29–30 days for 2.8 °C and 47–48 days for 0.5 °C (n = 3/incubator; 12/treatment). Unsedated larvae were placed laterally on the left side in a watch glass with 500µL of seawater set on a stage thermally controlled by a circulating cooling bath calibrated to the respective temperature group at 2.8 °C or 0.5 °C. Each larva was video recorded for one minute at 4× magnification under a stereomicroscope. Heart rate was determined by counting heartbeats within a 30 s time window when the larvae was not moving using a manual counter. Arrhythmia, a measure of the irregularity in the heartbeats, was calculated using the number of frames between beats for a 20 s period and the standard deviation between the first seven beats recorded^36^. All analysis was done blindly in reference to crude oil treatment and all recordings were analyzed by the same observer.

### Sampling of feeding larvae

Following confirmation of yolk sac absorption and initiation of first feeding in the additional control incubator at each temperature (day 52 at 2.8 °C and day 76 at 0.5 °C), 30 larvae were randomly sampled from each incubator, anesthetized, and photographed at 1.6× magnification. Information on larval length, swim bladder inflation (absence/presence), feeding success (absence/presence, with ‘0′ being an empty stomach and ‘1’ containing a single/ multiple food particles), and incidence of deformities was extracted from the images for each larva (Supplementary Table S4). Prevalence of deformities of the eyes, jaws, spine, and pericardial edema was quantified using absence (0)/ presence (1) for each larva. An index was calculated for each incubator using the number of fish with a given condition divided by the total number of fish sampled from each incubator^99^. The specific growth rate was calculated using incubator-averaged length measurements taken from pictures of yolk sac larvae following hatch (${\mathrm{L}}_{\mathrm{o}})$ and feeding larvae (${\mathrm{L}}_{\mathrm{t}})$ of each temperature group and accounting for the period between the time points (t) in the following equation:

3 $$\mathrm{Specific growth rate }(\mathrm{\% length }{\mathrm{day}}^{-1})=100*\frac{\mathrm{ln}{L}_{t}-\mathrm{ln}{L}_{o}}{t}$$

### Statistical analyses

Statistical analyses to assess the effect of increased temperature, crude oil exposure, and the interaction of these two stressors was performed in R^100^ using the ‘nlme’ package^101^. Water chemistry results were modeled using a simple linear model and a three-way interaction term (time, temperature, and oil exposure), and a Pearson’s correlation was run on initial concentrations. A linear mixed effect model (LME) was run for single measures in each incubator (e.g., survival, tPAH accumulation in embryos, gene expression, growth rate, and deformity scores). A generalized least squares linear model (GLS) was used for measurements on single organisms within an incubator (e.g., embryo specific gravity, length and yolk sac measurements, and heart rate and arrythmia measurements). The GLS model was run with a compound symmetric correlation within the incubator which assumes constant correlation between the embryos/larvae in the incubator but not between incubators and takes into account the possible inequality of variance in the observations^102^. Temperature and crude oil treatments were always treated as fixed factors in models to reveal nonlinear trends in concentration–response while ‘incubator’ was run as a random effect. To test the statistical significance of possible interactions between the stressors of temperature and crude oil treatment, we used log-likelihood ratio tests to compare a model with a fixed effect interaction against a model omitting the interaction to obtain the best model for each response^103^.

An ANOVA was run on the best model fit and reported together with the fitted model in the text of each figure. Coefficient estimates were extracted from models with significant interactions to assess whether these acted in an antagonistic or synergistic manner for each treatment combination^104^. An interaction coefficient estimate < 0 was categorized as congruent with stressor antagonism, a coefficient estimate > 0 was congruent with stressor synergism. Responses that did not have significant interaction terms when significant effects were present in both temperature and crude oil treatments revealed additive effects. The normality of model residuals was checked and non-normally distributed data (e.g., length and embryo specific gravity) were log-transformed. Treatment effects from all models are reported in the Supplementary Information (Supplementary Table S5), model parameters and ANOVA results (F-values and *p* values) are displayed in each figure. “Statistical significance” is not stated in the text to reduce the dichotomous interpretation of responses, rather, model outputs are reported, and trends are discussed in terms of biological significance^105,106^. Correlations were run for feeding larvae morphometrics and deformities using individual paired assessments and the non-parametric Spearman method. All effects reported in the Results are where *p* values < 0.05. Values are displayed as mean ± 1 standard error of the mean (SEM), and an arbitrary significance level was set at *p* values < 0.05.

## Supplementary Information

Supplementary Information 1.

Supplementary Information 2.

Supplementary Information 3.

## Data Availability

The data that support the findings of this study are available in DataverseNO with the DOI identifier “https://doi.org/10.18710/S94YFT”.

## References

[1] IPCC. *The Ocean and Cryosphere in a changing Climate—Summary for Policymakers* (2019).

[2] Carmack E (2015). Toward quantifying the increasing role of oceanic heat in sea ice loss in the new arctic. Bull. Am. Meteorol. Soc..

[3] Crain CM, Kroeker K, Halpern BS (2008). Interactive and cumulative effects of multiple human stressors in marine systems. Ecol. Lett..

[4] Borgå K (2019). The Arctic ecosystem: a canary in the coal mine for global multiple stressors. Environ. Toxicol. Chem..

[5] Lind S, Ingvaldsen RB, Furevik T (2018). Arctic warming hotspot in the northern Barents Sea linked to declining sea-ice import. Nat. Clim. Change.

[6] Onarheim IH, Eldevik T, Smedsrud LH, Stroeve JC (2018). Seasonal and regional manifestation of Arctic Sea ice loss. J. Clim..

[7] Screen JA, Simmonds I (2010). Increasing fall-winter energy loss from the Arctic Ocean and its role in Arctic temperature amplification. Geophys. Res. Lett..

[8] Onarheim IH, Årthun M (2017). Toward an ice-free Barents Sea. Geophys. Res. Lett..

[9] Champine, R. D., Morris, R. & Elder, S. The melting Arctic is now open for business. *National Geographic Magazine* (2019).

[10] Orourke R (2020). Changes in the Arctic: Background and Issues for Congress.

[11] Eriksen E, Huserbråten M, Gjøsæter H, Vikebø F, Albretsen J (2019). Polar cod egg and larval drift patterns in the Svalbard archipelago. Polar Biol..

[12] Eguíluz VM, Fernández-Gracia J, Irigoien X, Duarte CM (2016). A quantitative assessment of Arctic shipping in 2010–2014. Sci. Rep..

[13] Ellis, B., & Brigham, L. *Arctic Marine Shipping Assessment 2009 Report*. (2009).

[14] Pörtner H-O, Farrell AP (2008). Physiology and climate change. Science.

[15] Pollino CA, Holdway DA (2002). Toxicity testing of crude oil and related compounds using early life stages of the crimson-spotted rainbowfish (*Melanotaenia fluviatilis*). Ecotoxicol. Environ. Saf..

[16] Miller B, Kendall AW (2009). Early Life History of Marine Fishes.

[17] Dahlke FT (2018). Northern cod species face spawning habitat losses if global warming exceeds 1.5°C. Sci. Adv..

[18] Petersen GI, Kristensen P (1998). Bioaccumulation of lipophilic substances in fish early life stages. Environ. Toxicol. Chem..

[19] Dahlke FT, Wohlrab S, Butzin M, Pörtner H-O (2020). Thermal bottlenecks in the life cycle define climate vulnerability of fish. Science.

[20] Jung J-H (2015). Differential toxicokinetics determines the sensitivity of two marine embryonic fish exposed to Iranian heavy crude oil. Environ. Sci. Technol..

[21] Ingvarsdóttir A (2012). Effects of different concentrations of crude oil on first feeding larvae of Atlantic herring (*Clupea harengus*). J. Mar. Syst..

[22] Pasparakis C, Esbaugh AJ, Burggren W, Grosell M (2019). Physiological impacts of deepwater horizon oil on fish. Comp. Biochem. Physiol. Part C Toxicol. Pharmacol..

[23] Steiner NS (2019). Impacts of the changing ocean-sea ice system on the key forage fish arctic cod (*Boreogadus saida*) and subsistence fisheries in the western Canadian arctic—evaluating linked climate, ecosystem and economic (CEE) models. Front. Mar. Sci..

[24] Kortsch S, Primicerio R, Fossheim M, Dolgov AV, Aschan M (2015). Climate change alters the structure of arctic marine food webs due to poleward shifts of boreal generalists. Proc. R. Soc. B Biol. Sci..

[25] Harter BB, Elliott KH, Divoky GJ, Davoren GK (2013). Arctic cod (*Boreogadus saida*) as prey: fish length-energetics relationships in the Beaufort Sea and Hudson Bay. Arctic.

[26] Graham M, Hop H (1995). Aspects of reproduction and larval biology of Arctic cod (*Boreogadus saida*). Arctic.

[27] Gradinger RR, Bluhm BA (2004). In-situ observations on the distribution and behavior of amphipods and Arctic cod (*Boreogadus saida*) under the sea ice of the High Arctic Canada Basin. Polar Biol..

[28] Laurel BJ, Copeman LA, Spencer M, Iseri P (2018). Comparative effects of temperature on rates of development and survival of eggs and yolk-sac larvae of Arctic cod (*Boreogadus saida*) and walleye pollock (*Gadus chalcogrammus*). ICES J. Mar. Sci..

[29] ICES. *Report of the Arctic Fisheries Working Group*. 859 http://www.ices.dk/sites/pub/Publication%20Reports/Expert%20Group%20Report/acom/2018/AFWG/00-AFWG%202018%20Report.pdf (2018).

[30] Eriksen E, Ingvaldsen RB, Nedreaas K, Prozorkevich D (2015). The effect of recent warming on polar cod and beaked redfish juveniles in the Barents Sea. Reg. Stud. Mar. Sci..

[31] Astthorsson OS (2016). Distribution, abundance and biology of polar cod, *Boreogadus saida*, in Iceland–East Greenland waters. Polar Biol..

[32] Divoky GJ, Lukacs PM, Druckenmiller ML (2015). Effects of recent decreases in arctic sea ice on an ice-associated marine bird. Prog. Oceanogr..

[33] Hansen MO, Nielsen TG, Stedmon CA, Munk P (2012). Oceanographic regime shift during 1997 in Disko Bay, Western Greenland. Limnol. Oceanogr..

[34] Nahrgang J (2014). Gender specific reproductive strategies of an Arctic key species (*Boreogadus saida*) and implications of climate change. PLoS ONE.

[35] Huserbråten MBO, Eriksen E, Gjøsæter H, Vikebø F (2019). Polar cod in jeopardy under the retreating Arctic sea ice. Commun. Biol..

[36] Nahrgang J (2016). Early life stages of an arctic keystone species (*Boreogadus saida*) show high sensitivity to a water-soluble fraction of crude oil. Environ. Pollut..

[37] Laurel BJ (2019). Embryonic crude oil exposure impairs growth and lipid allocation in a keystone arctic forage fish. iScience.

[38] Politis SN (2017). Temperature effects on gene expression and morphological development of European eel, *Anguilla anguilla* larvae. PLoS ONE.

[39] O’Dea RE, Lagisz M, Hendry AP, Nakagawa S (2019). Developmental temperature affects phenotypic means and variability: a meta-analysis of fish data. Fish Fish..

[40] Réalis-Doyelle E, Pasquet A, De Charleroy D, Fontaine P, Teletchea F (2016). Strong effects of temperature on the early life stages of a cold stenothermal fish species, brown trout (*Salmo trutta* L.). PLoS ONE.

[41] Réalis-Doyelle E, Pasquet A, Fontaine P, Teletchea F (2018). How climate change may affect the early life stages of one of the most common freshwater fish species worldwide: the common carp (*Cyprinus carpio*). Hydrobiologia.

[42] Hicken CE (2011). Sublethal exposure to crude oil during embryonic development alters cardiac morphology and reduces aerobic capacity in adult fish. Proc. Natl. Acad. Sci..

[43] Carls MG, Rice SD, Hose JE (1999). Sensitivity of fish embryos to weathered crude oil: Part I. Low-level exposure during incubation causes malformations, genetic damage, and mortality in larval pacific herring (*Clupea pallasi*). Environ. Toxicol. Chem..

[44] Incardona JP (2017). Molecular mechanisms of crude oil developmental toxicity in fish. Arch. Environ. Contam. Toxicol..

[45] Sørhus E (2017). Novel adverse outcome pathways revealed by chemical genetics in a developing marine fish. Elife.

[46] Incardona JP, Scholz NL (2016). The influence of heart developmental anatomy on cardiotoxicity-based adverse outcome pathways in fish. Aquat. Toxicol..

[47] Perrichon P (2018). Combined effects of elevated temperature and Deepwater Horizon oil exposure on the cardiac performance of larval mahi–mahi, *Coryphaena hippurus*. PLoS ONE.

[48] Pasparakis C (2017). Combined effects of oil exposure, temperature and ultraviolet radiation on buoyancy and oxygen consumption of embryonic mahi–mahi, *Coryphaena hippurus*. Aquat. Toxicol..

[49] Pasparakis C, Mager EM, Stieglitz JD, Benetti D, Grosell M (2016). Effects of Deepwater Horizon crude oil exposure, temperature and developmental stage on oxygen consumption of embryonic and larval mahi–mahi (*Coryphaena hippurus*). Aquat. Toxicol..

[50] Gunderson AR, Armstrong EJ, Stillman JH (2016). Multiple stressors in a changing world: the need for an improved perspective on physiological responses to the dynamic marine environment. Annu. Rev. Mar. Sci..

[51] McNicholl DG, Davoren GK, Majewski AR, Reist JD (2018). Isotopic niche overlap between co-occurring capelin (*Mallotus villosus*) and polar cod (*Boreogadus saida*) and the effect of lipid extraction on stable isotope ratios. Polar Biol..

[52] Kühn S (2018). Plastic ingestion by juvenile polar cod (*Boreogadus saida*) in the Arctic Ocean. Polar Biol..

[53] Bouchard C, Fortier L (2011). Circum-arctic comparison of the hatching season of polar cod *Boreogadus saida*: a test of the freshwater winter refuge hypothesis. Prog. Oceanogr..

[54] Laurel BJ, Spencer M, Iseri P, Copeman LA (2016). Temperature-dependent growth and behavior of juvenile Arctic cod (*Boreogadus saida*) and co-occurring North Pacific gadids. Polar Biol..

[55] Drost HE (2016). Upper thermal limits of the hearts of Arctic cod *Boreogadus saida* : adults compared with larvae: *boreogadus saida* thermal limits. J. Fish Biol..

[56] Bender ML (2016). Effects of chronic dietary petroleum exposure on reproductive development in polar cod (*Boreogadus saida*). Aquat. Toxicol..

[57] Bender ML (2018). Effects of acute exposure to dispersed oil and burned oil residue on long-term survival, growth, and reproductive development in polar cod (*Boreogadus saida*). Mar. Environ. Res..

[58] Boehm PD, Neff JM, Page DS (2007). Assessment of polycyclic aromatic hydrocarbon exposure in the waters of Prince William Sound after the Exxon Valdez oil spill: 1989–2005. Mar. Pollut. Bull..

[59] Sammarco PW (2013). Distribution and concentrations of petroleum hydrocarbons associated with the BP/Deepwater Horizon Oil Spill, Gulf of Mexico. Mar. Pollut. Bull..

[60] Berenshtein I (2020). Invisible oil beyond the *Deepwater Horizon* satellite footprint. Sci. Adv..

[61] Incardona JP (2009). Cardiac arrhythmia is the primary response of embryonic pacific herring (*Clupea pallasi*) exposed to crude oil during weathering. Environ. Sci. Technol..

[62] Incardona JP (2013). Exxon Valdez to Deepwater Horizon: comparable toxicity of both crude oils to fish early life stages. Aquat. Toxicol. Amst. Neth..

[63] de Soysa TY (2012). Macondo crude oil from the Deepwater Horizon oil spill disrupts specific developmental processes during zebrafish embryogenesis. BMC Biol..

[64] Incardona JP (2015). Very low embryonic crude oil exposures cause lasting cardiac defects in salmon and herring. Sci. Rep..

[65] Heintz RA (2000). Delayed effects on growth and marine survival of pink salmon *Oncorhynchus gorbuscha* after exposure to crude oil during embryonic development. Mar. Ecol. Prog. Ser..

[66] Sorheim, K. R. & Moldestad, M. O. *Weathering properties of the Goliat Kobbe and two Goliat Blend of Kobbe and Realgrunnen crude oils*. (2008).

[67] Sørensen L, Melbye AG, Booth AM (2014). Oil droplet interaction with suspended sediment in the seawater column: influence of physical parameters and chemical dispersants. Mar. Pollut. Bull..

[68] Sørensen L (2019). Accumulation and toxicity of monoaromatic petroleum hydrocarbons in early life stages of cod and haddock. Environ. Pollut..

[69] Meador JP, Nahrgang J (2019). Characterizing crude oil toxicity to early-life stage fish based on a complex mixture: Are we making unsupported assumptions?. Environ. Sci. Technol..

[70] Sørensen L (2017). Oil droplet fouling and differential toxicokinetics of polycyclic aromatic hydrocarbons in embryos of Atlantic haddock and cod. PLoS ONE.

[71] Carls MG (2008). Fish embryos are damaged by dissolved PAHs, not oil particles. Aquat. Toxicol..

[72] Hansen BH (2019). Developmental effects in fish embryos exposed to oil dispersions—the impact of crude oil micro-droplets. Mar. Environ. Res..

[73] Olsvik PA, Berntssen MHG, Hylland K, Eriksen DØ, Holen E (2012). Low impact of exposure to environmentally relevant doses of 226Ra in Atlantic cod (*Gadus morhua*) embryonic cells. J. Environ. Radioact..

[74] Sundby S, Kristiansen T (2015). The principles of buoyancy in marine fish eggs and their vertical distributions across the world oceans. PLoS ONE.

[75] Spencer ML, Vestfals CD, Mueter FJ, Laurel BJ (2020). Ontogenetic changes in the buoyancy and salinity tolerance of eggs and larvae of polar cod (*Boreogadus saida*) and other gadids. Polar Biol..

[76] Pasparakis C, Wang Y, Stieglitz JD, Benetti DD, Grosell M (2019). Embryonic buoyancy control as a mechanism of ultraviolet radiation avoidance. Sci. Total Environ..

[77] Kent D, Drost HE, Fisher J, Oyama T, Farrell AP (2016). Laboratory rearing of wild Arctic cod *Boreogadus saida* from egg to adulthood: rearing *boreogadus saida* from egg to adulthood. J. Fish Biol..

[78] Jordaan A, Hayhurst SE, Kling LJ (2006). The influence of temperature on the stage at hatch of laboratory reared *Gadus morhua* and implications for comparisons of length and morphology. J. Fish Biol..

[79] Porter SM, Bailey KM (2007). The effect of early and late hatching on the escape response of walleye pollock (*Theragra chalcogramma*) larvae. J. Plankton Res..

[80] Spicer JI, Tills O, Truebano M, Rundle SD, Burggren W, Dubansky B (2018). Developmental plasticity and heterokairy. Development and Environment.

[81] Bouchard C (2017). Climate warming enhances polar cod recruitment, at least transiently. Prog. Oceanogr..

[82] Koenker BL, Laurel BJ, Copeman LA, Ciannelli L (2018). Effects of temperature and food availability on the survival and growth of larval Arctic cod (*Boreogadus saida*) and walleye pollock (*Gadus chalcogrammus*). ICES J. Mar. Sci..

[83] Bouchard C, Fortier L (2020). The importance of *Calanus glacialis* for the feeding success of young polar cod: a circumpolar synthesis. Polar Biol..

[84] Balazy K, Trudnowska E, Wichorowski M, Błachowiak-Samołyk K (2018). Large versus small zooplankton in relation to temperature in the Arctic shelf region. Polar Res..

[85] Weydmann A (2014). Shift towards the dominance of boreal species in the Arctic: inter-annual and spatial zooplankton variability in the West Spitsbergen Current. Mar. Ecol. Prog. Ser..

[86] Marsh JM, Mueter FJ, Quinn TJ (2019). Environmental and biological influences on the distribution and population dynamics of polar cod (*Boreogadus saida*) in the US Chukchi Sea. Polar Biol..

[87] Lange R, Marshall D (2017). Ecologically relevant levels of multiple, common marine stressors suggest antagonistic effects. Sci. Rep..

[88] Liess M, Foit K, Knillmann S, Schäfer RB, Liess H-D (2016). Predicting the synergy of multiple stress effects. Sci. Rep..

[89] du Sert NP (2020). Reporting animal research: explanation and elaboration for the ARRIVE guidelines 2.0. PLoS Biol..

[90] Holst JC, McDonald A (2000). FISH-LIFT: a device for sampling live fish with trawls. Fish. Res..

[91] Hall TE, Smith P, Johnston IA (2004). Stages of embryonic development in the Atlantic cod *Gadus morhua*. J. Morphol..

[92] Houde ED, Fuiman LA (1989). Mortality. Fishery Science.

[93] Sørensen L, Silva MS, Booth AM, Meier S (2016). Optimization and comparison of miniaturized extraction techniques for PAHs from crude oil exposed Atlantic cod and haddock eggs. Anal. Bioanal. Chem..

[94] Sørensen L, Meier S, Mjøs SA (2016). Application of gas chromatography/tandem mass spectrometry to determine a wide range of petrogenic alkylated polycyclic aromatic hydrocarbons in biotic samples. Rapid Commun. Mass Spectrom..

[95] Pfaffl MW (2001). A new mathematical model for relative quantification in real-time RT-PCR. Nucleic Acids Res..

[96] Riley P, Skirrow G (1975). Chemical Oceanography.

[97] Laurel BJ, Copeman LA, Hurst TP, Parrish CC (2010). The ecological significance of lipid/fatty acid synthesis in developing eggs and newly hatched larvae of Pacific cod (*Gadus macrocephalus*). Mar. Biol..

[98] Schneider CA, Rasband WS, Eliceiri KW (2012). NIH Image to ImageJ: 25 years of image analysis. Nat. Methods.

[99] Wassenberg DM, Di Giulio RT (2004). Synergistic embryotoxicity of polycyclic aromatic hydrocarbon aryl hydrocarbon receptor agonists with cytochrome P4501A inhibitors in *Fundulus heteroclitus*. Environ. Health Perspect..

[100] R Core Team. *R: A Language and Environment for Statistical Computing.* (R Foundation for Statistical Computing, version 2018). https://www.R-project.org/.

[101] Pinheiro, J., Bates, D., DebRoy, S., Sarkar, D. & Van Willigen, B. *nlme: Linear and Nonlinear Mixed Effects Models*. (2020).

[102] Pinheiro, J. & Bates, D. Fitting linear mixed-effects models. In *Mixed-Effects Models in S and S-Plus* 133–199 (Springer, 2000).

[103] Zuur A, Ieno EN, Walker N, Saveliev AA, Smith GM (2009). Mixed Effects Models and Extensions in Ecology with R.

[104] Folt CL, Chen CY, Moore MV, Burnaford J (1999). Synergism and antagonism among multiple stressors. Limnol. Oceanogr..

[105] Wasserstein RL, Schirm AL, Lazar NA (2019). Moving to a world beyond “p < 0.05”. Am. Stat..

[106] Amrheim V, Greenland S, McShane B (2019). Time to retire statistical significance Nature2019.pdf. Nature.

