# Supplementary material for: Combined effects of crude oil exposure and warming on eggs and larvae of an arctic forage fish
Source: Sci Rep. 2021 Apr 16;11:8410. doi: 10.1038/s41598-021-87932-2 (PMC8052424; doi:10.1038/s41598-021-87932-2)
Supplement: Supplementary file 1 — Supplementary Information 1. [file 41598_2021_87932_MOESM1_ESM.pdf]

Supplementary Information

for

Manuscript: Combined effects of crude oil exposure and warming on eggs and larvae of an Arctic forage fish

Morgan Lizabeth Bender[1*], Julia Giebichenstein[1], Ragnar N Teisrud[1], Jennifer Laurent [1], Marianne Frantzen[2], James P Meador[3], Lisbet Sørensen[4], Bjørn Henrik Hansen[4], Helena C Reinardy [5,6], Benjamin Laurel[7], Jasmine M Nahrgang[1]

[1] Department of Arctic and Marine Biology, UiT The Arctic University of Norway, 9037 Tromsø, Norway

[2] Akvaplan-niva, Fram Centre, 9296 Tromsø, Norway

[3] Environmental and Fisheries Sciences Division, Northwest Fisheries Science Center, National Marine Fisheries Service, National Oceanic and Atmospheric Administration, 2725 Montlake Blvd. East, Seattle, Washington 98112

[4] SINTEF Ocean, Environment and New Resources, 7465 Trondheim, Norway

[5] Scottish Association for Marine Science, Oban PA37 1QA, UK

[6] Department of Arctic Technology, The University Centre in Svalbard, Longyearbyen, Svalbard

[7] Fisheries Behavioral Ecology Program, Alaska Fisheries Science Center, National Marine Fisheries Service, NOAA, Hatfield Marine Science Center, Newport, OR 97365, USA

[*] Corresponding author: morgan.l.bender@uit.no

Table S1. Concentrations (ng/L) of the 44 PAH analytes measured in the water at the start of the exposure (Day 0) for each temperature and crude oil treatment group. Lower limit of detection (LOD) is noted for each analyte in the first column.

**Analyte (ng/L)**	**LOD**	**0.5°C**				**2.8°C**			
		**Control**	**Low**	**Medium**	**High**	**Control**	**Low**	**Medium**	**High**
**Benzothiophene**	0.07	0.10	0.13	0.18	0.21	0.14	0.10	0.12	0.15
**Naphthalene**	0.39	0.81	0.74	1.80	14.27	0.74	0.89	1.37	12.39
**C1-NAP**	0.05	0.10	0.27	1.90	16.84	0.09	0.53	1.34	16.47
**C2-NAP**	1.13	<LOD	<LOD	2.09	12.46	<LOD	<LOD	1.43	12.19
**C3-NAP**	0.02	0.05	0.41	6.04	20.10	0.06	0.53	1.52	31.62
**C4-NAP**	0.04	0.07	0.67	16.93	23.88	0.12	0.57	2.06	64.19
**Biphenyl**	0.13	0.24	0.26	0.68	4.00	0.21	0.40	0.67	3.16
**Acenaphthylene**	0.10	0.10	<LOD	0.40	1.25	0.11	0.13	0.23	1.98
**Acenapthene**	0.04	0.05	<LOD	0.17	0.57	0.05	<LOD	0.10	0.56
**Dibenzofuran**	0.15	0.19	0.21	0.61	2.87	0.20	0.29	0.49	2.41
**Fluorene**	0.12	0.14	0.20	0.73	3.78	0.15	0.28	0.54	3.06
**C1-FLU**	0.01	0.01	0.05	0.55	1.56	0.02	0.07	0.17	2.32
**C2-FLU**	0.01	0.02	0.14	3.36	3.28	0.03	0.10	0.34	9.85
**C3-FLU**	0.06	<LOD	0.07	2.27	1.08	<LOD	<LOD	0.13	5.20
**Phenanthrene**	0.14	0.24	0.72	4.54	14.22	0.82	0.84	1.89	15.41
**Anthracene**	0.88	<LOD	<LOD	0.97	<LOD	<LOD	<LOD	<LOD	2.71
**C1-PHE**	0.01	0.02	0.15	2.79	4.36	0.02	0.13	0.45	9.72
**C2-PHE**	0.25	<LOD	0.29	3.98	3.10	<LOD	0.26	0.59	6.48
**C3-PHE**	0.01	0.01	0.35	8.46	2.67	0.02	0.12	0.40	10.01
**C4-PHE**	0.12	<LOD	0.31	8.74	1.81	<LOD	<LOD	0.21	9.10
**Dibenzothiophene**	0.11	0.11	0.18	1.31	2.05	0.13	0.21	0.36	4.27
**C1-DBT**	1.76	<LOD	<LOD	<LOD	<LOD	<LOD	<LOD	<LOD	<LOD
**C2-DBT**	0.01	0.01	0.02	0.23	0.18	0.01	0.01	0.03	0.36
**C3-DBT**	0.01	<LOD	0.01	0.34	0.11	<LOD	0.01	0.02	0.40
**C4-DBT**	0.00	<LOD	0.01	0.25	0.06	0.00	0.00	0.01	0.26
**Fluoranthene**	0.22	0.23	0.35	0.79	0.76	0.25	<LOD	0.24	1.02
**Pyrene**	0.02	0.10	0.11	1.20	0.95	0.16	0.08	0.17	1.70
**C1-FLA/PYR**	0.01	0.01	0.10	1.50	1.08	0.02	0.04	0.16	2.18
**C2-FLA/PYR**	0.01	<LOD	0.07	1.06	0.44	<LOD	0.02	0.05	1.38
**C3-FLA/PYR**	0.01	<LOD	0.06	1.03	0.35	0.01	0.01	0.04	1.12
**Benz[a]anthracene**	0.06	<LOD	<LOD	0.78	0.11	<LOD	<LOD	<LOD	0.79
**Chrysene**	0.00	<LOD	0.05	2.67	0.92	<LOD	<LOD	0.07	2.76
**C1-CHR**	0.01	<LOD	0.04	0.61	0.19	<LOD	<LOD	0.03	0.70
**C2-CHR**	0.03	<LOD	<LOD	0.08	<LOD	<LOD	<LOD	<LOD	0.09
**C3-CHR**	0.01	<LOD	0.01	0.08	0.02	<LOD	0.02	<LOD	0.09
**C4-CHR**	0.02	<LOD	<LOD	0.07	<LOD	<LOD	<LOD	<LOD	0.08
**Benzo[b]fluoranthene**	0.03	<LOD	<LOD	0.35	0.11	<LOD	<LOD	<LOD	0.29
**Benzo[k]fluoranthene**	0.00	<LOD	<LOD	0.25	<LOD	<LOD	<LOD	<LOD	0.21
**Benzo[e]pyrene**	0.00	<LOD	<LOD	0.46	0.07	<LOD	<LOD	<LOD	0.37
**Benzo[a]pyrene**	0.00	<LOD	<LOD	0.28	<LOD	<LOD	<LOD	<LOD	<LOD
**Perylene**	0.00	<LOD	<LOD	0.12	<LOD	<LOD	<LOD	<LOD	0.06
**Indeno[1,2,3-cd]pyrene**	0.07	<LOD	<LOD	<LOD	<LOD	<LOD	<LOD	<LOD	<LOD
**Dibenz[ah]anthracene**	0.18	<LOD	<LOD	<LOD	<LOD	<LOD	<LOD	<LOD	<LOD
**Benzo[ghi]perylene**	0.00	<LOD	<LOD	0.11	<LOD	<LOD	<LOD	<LOD	0.11
**Sum PAH**		2.60	5.99	80.78	139.70	3.34	5.65	15.22	237.26
**Sum NAP**		1.03	2.10	28.76	87.55	1.01	2.52	7.72	136.86
**Sum 2-3rings PAH**		1.23	3.09	40.57	47.16	1.89	2.95	6.74	87.42
**Sum 4-6rings PAH**		0.34	0.81	11.45	4.99	0.44	0.17	0.76	12.98

Table S2a. Concentrations (ng/g wet weight) of the 44 PAH analytes measured in embryos at Day 4 for each temperature and crude oil treatment group.
See attached Excel file “S2C Table S2.xlsx”

Table S2b. Concentrations (ng/g wet weight) of the 44 PAH analytes measured in embryos at Day18 for each temperature and crude oil treatment group.
See attached Excel file “S2C Table S2.xlsx”

Table S3. Stage definition for polar cod embryos and larvae in the present work. Degree day duration for each stage in each temperature group is calculated in the right columns.

Stage	Phenology	Description	Degree days for stage duration	
			0.5°C	2.8°C
Cleavage Stage	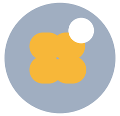	Initiated with fertilization, synchronous cell division until 128 cell stage	0 - 2	0 - 8.4
Blastulation	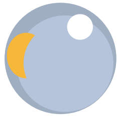	Blastodisc present on top of yolk	2 - 3.5	8.4 - 11.2
Gastrulation	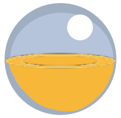	Appearance of germ ring; graduated stages of epiboly; end of gastrulation identified with 50% epiboly	3.5 - 4.5	11.2 - 16.8
Organogenesis	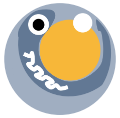	Formation of visible notochord and somites; eyes become pigmented; heart beating visible; embryos move inside chorion; hatch glands appear; 100% tail curl; body pigmentation visible	4.5 – 31.0*	16.8 - 84.0*
Hatch	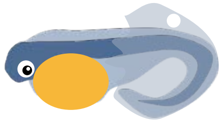	The event of embryos exiting chorion, hatch period defined by first observation on unperturbed hatching in incubator and ends with last viable eggs hatching while at incubator surface	21 - 32.5	78.4 - 89.6
Yolk Sac	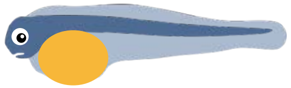	Free larvae with yolk sac; undeveloped digestive tract, jaws, eyes, and face; finfold present; swim bladder inflation, begin feeding on rotifers and artemia nauplii	31 - 35.5	84.0 - 137.2
Exogenous feeding larvae**	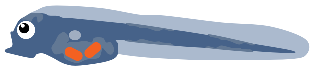	Yolk sac absorbed; exogenously feeding on artemia; straighten notochord; no separate medial fins; caudal fin rays begin to form; fully developed jaws, eyes, and face; more pigmentation in eyes and body	35.5 - 86	137.2 - 324.8

Based of *G. morhau* stages in Hall et al. 2004; Miller and Kendall 2009;

* Organogenesis stageduration calculated until peak hatch at each temperature

**Exogenous feeding stage duration based off data not presented in this study

Table S4. Overview of morphometrics characteristics used to determine phenotype of yolk sac and feeding larvae. Black arrows demark the referred morphological alteration.

Condition	0- Absent	1- Present	Explanation
Yolk sac edema	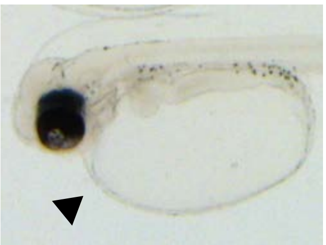	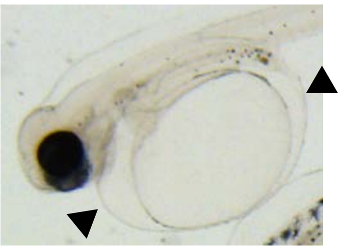	Excessive space around the yolk sac
Pericardial edema	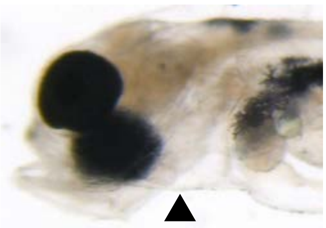	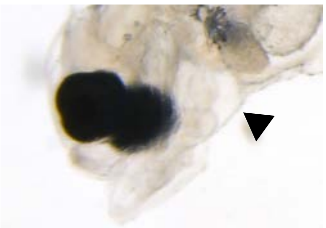	pericardial membrane not in contact with heart chambers, excessive fluid around heart
Eye deformities	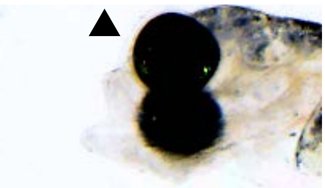	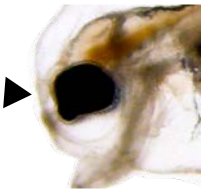	Non-round eyes visible
Jaw deformities	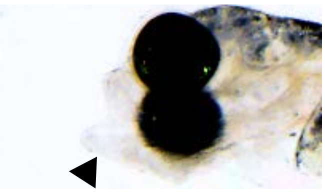	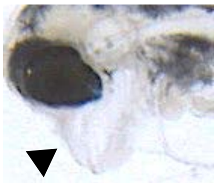	Jaw distended or absent
Feeding success	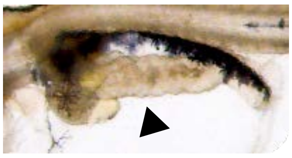	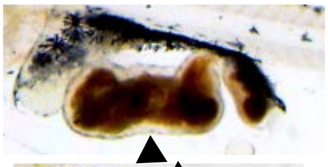	Stomach containing one or mor food particles
Swim bladder inflation	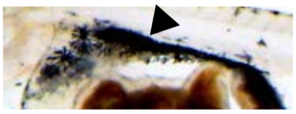	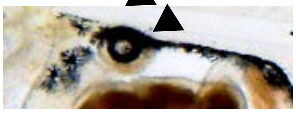	Inflated swim bladder visible
Spinal deformities	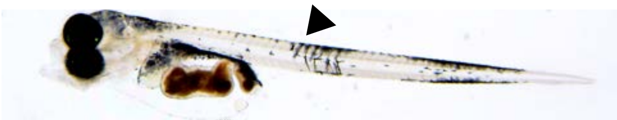		0- Absent
	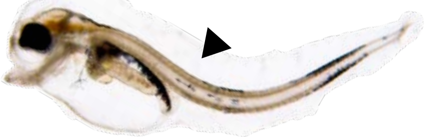		1- Present, clear curvature of spine

Table S5. Best model summaries for investigated responses. Significant factors are bolded and interaction types are noted when the interaction was included in the best model.

Attached Excel File "S2C Table S5.xlsx"

Table S6. Summary of response interactions for each of the 22 responses investigated in this study. Interaction presence and type was determined using the log likelihood method and the strength of the interaction is determined from its respective p-value in an ANOVA test (strong interactions have p-values <0.05). Bold responses are those with strong interactions.

LIFE STAGE	RESPONSE	INTERACTION TYPE	STRENGTH
EMBRYONIC	Early embryonic stage mortality rate	Antagonistic	Weak
	Late embryonic stage mortality rate	Synergistic / Antagonistic	Weak
	***cyp1a* mRNA levels**	**Synergistic**	**Strong**
	hsp70 mRNA levels	None	
	hsp8 mRNA levels	Synergistic	Weak
	***cyp1c1* mRNA levels**	**Synergistic**	**Strong**
	**Embryo specific gravity**	**Antagonistic**	**Strong**
YOLK SAC LARVAE	**Yolk sac larvae mortality rate**	**Synergistic**	**Strong**
	Log (Length)	Synergistic / Antagonistic	Weak
	Yolk sac area	None	
	Heart rate	Synergistic / Antagonistic	Weak
	Heart arrhythmia	None	
FIRST FEEDING LARVAE	**Feeding larvae mortality rate**	**Synergistic**	**Strong**
	Yolk sac edema	None	
	Log (Length)	Synergistic / Antagonistic	Weak
	Specific growth rate	None	
	Swim bladder inflation	None	
	Feeding success	None	
	**Pericardial edema**	**Antagonistic**	**Strong**
	Eye deformities	None	
	**Jaw deformities**	**Synergistic**	**Strong**
	**Spinal deformities**	**Synergistic**	**Strong**

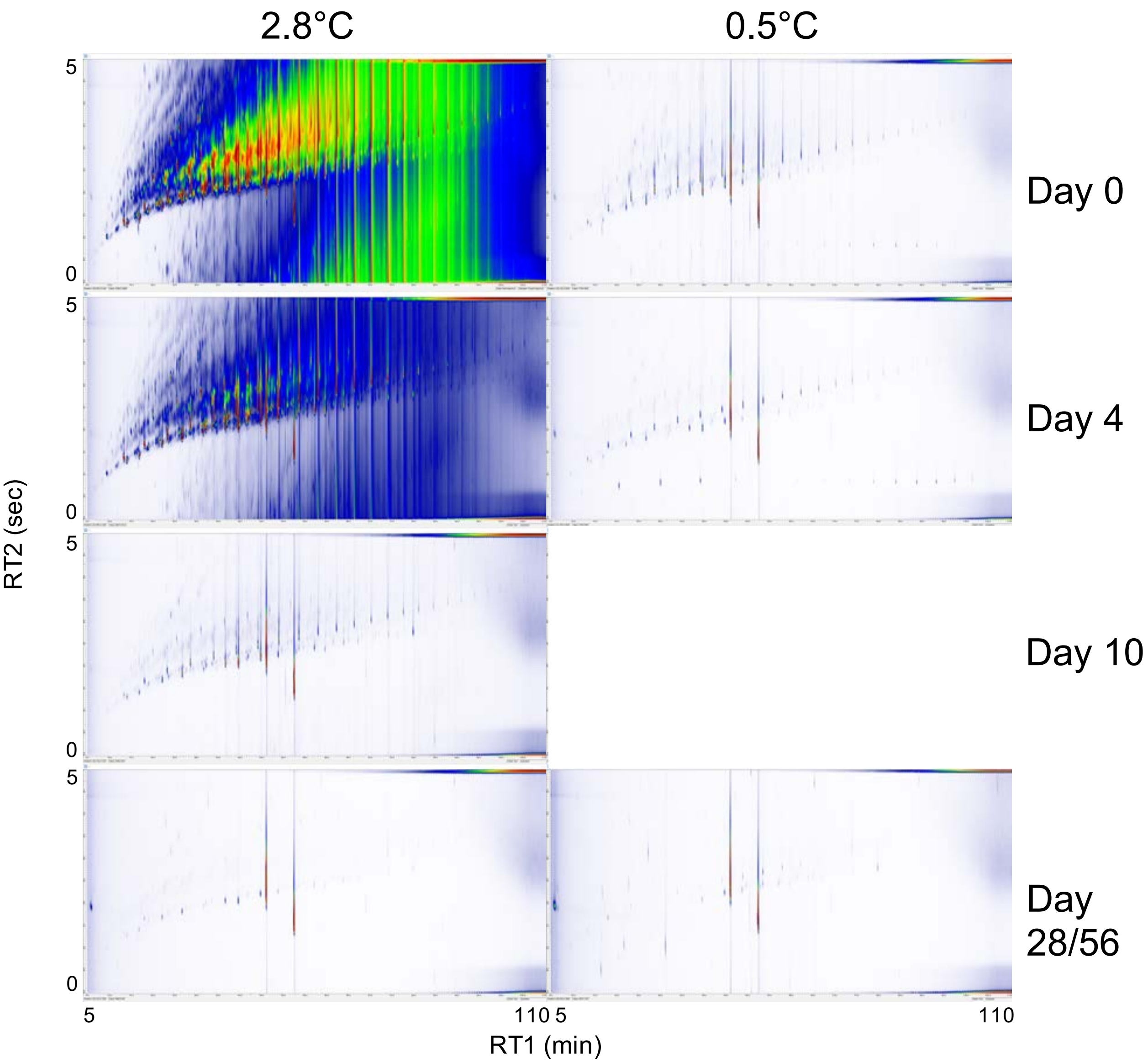

Figure S1. Two dimensional gas chromatography (GCxGC-MS) total ion chromatograms for water samples from the high oil treatments at four different timepoints. The two major peaks visible in all the chromatograms are internal standards. PAHs are found in the upper left corner and lower middle left, aliphatic compounds make up the prominent curved line across the middle, and finally monoaromatic compounds are between the upper group of PAHs and the aliphatic compounds. Color scheme is the relative signal strength from cool to warm.

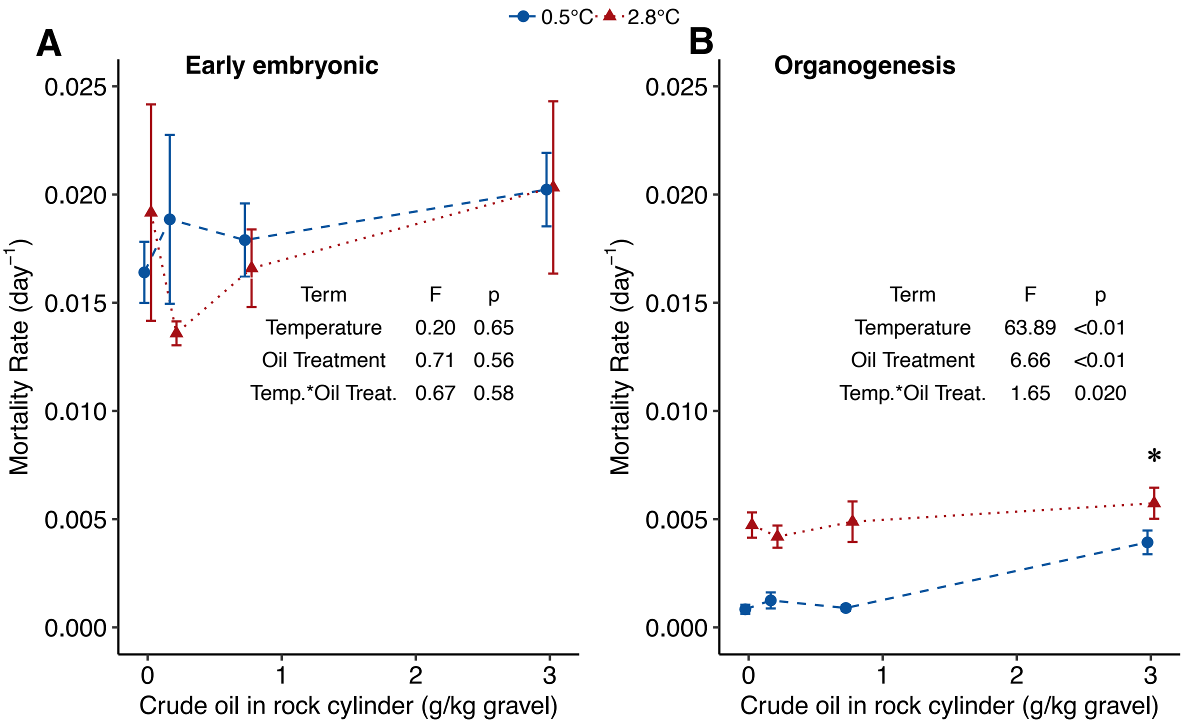

Figure S2. The effect of temperature and crude oil WSF exposure on instantaneous mortality rates for (A) early embryogenesis (day 0-5(2.8°C)/8(0.5°C)) and (B) late embryogenesis / organogenesis (day 6-43(2.8°C); 9-65(0.5°C)). Data is displayed as treatment means (± SEM depicted as bars, each treatment is represented by four replicate incubators). Colors and symbols indicate temperature groups and dashed and dotted lines represent the trend line for each temperature group. The terms of the best LME models for each gene are displayed in the respective panels with the associated ANOVA test F-values and p-value. An asterisk (*) above a treatment group indicates a statistically significant difference from the unexposed group.

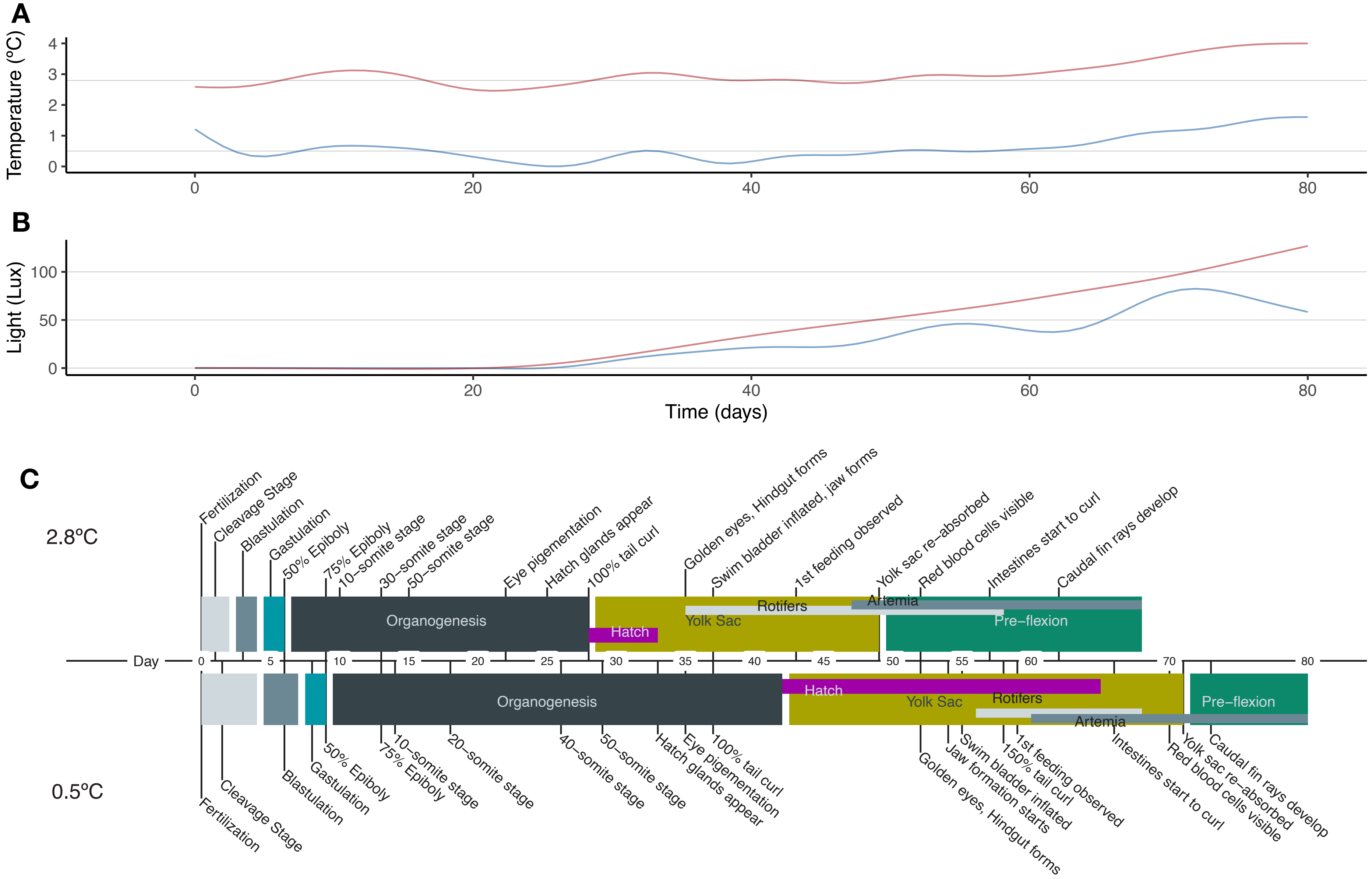

Figure S3. Timeline of the (A) temperature and (B) light levels in lux plotted as the smoothed line of mean daily averages from two incubators at

each temperature and lastly, (C) key developmental events in polar cod early life stages reared at the two temperatures for the full period of the present study. Milestone timing was based off daily imaging from additional control incubators. Feeding schemes are presented as small bars to the outer periphery of the stage bar at each temperature group. Stages were based off *G. mohua* staging presented in Hall *et al.* 2004.

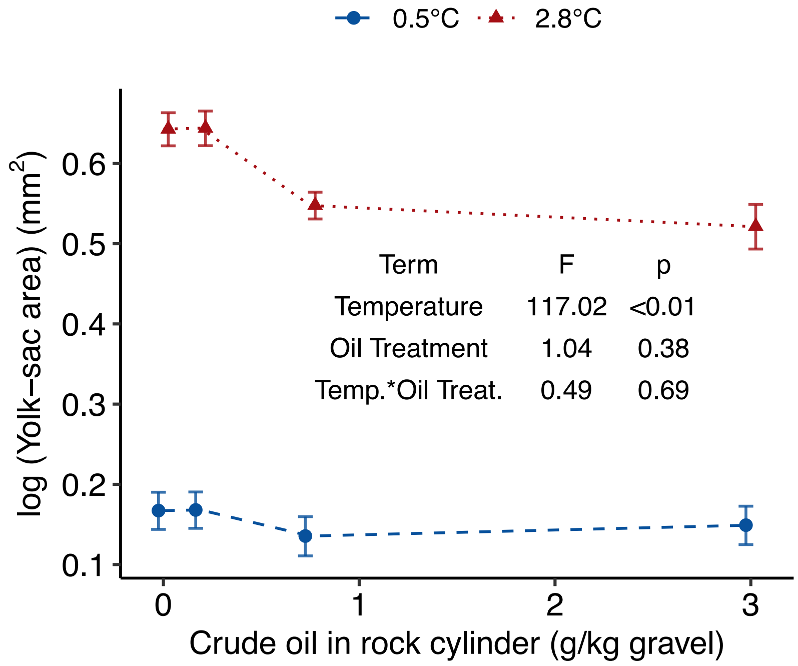

Figure S4. The effect of temperature and crude oil WSF exposure on yolk sac area (log scale) in yolk sac larvae at day 28 for 2.8°C and day 50 for 0.5°C displayed as treatment means (± SEM depicted as bars, each point includes 4 incubator means calculated from 20-30 larvae). Colors, symbols, and line type distinguish the temperature groups. The terms of the GLS model for yolk-sac area is displayed with the associated ANOVA test F-values and p-values.

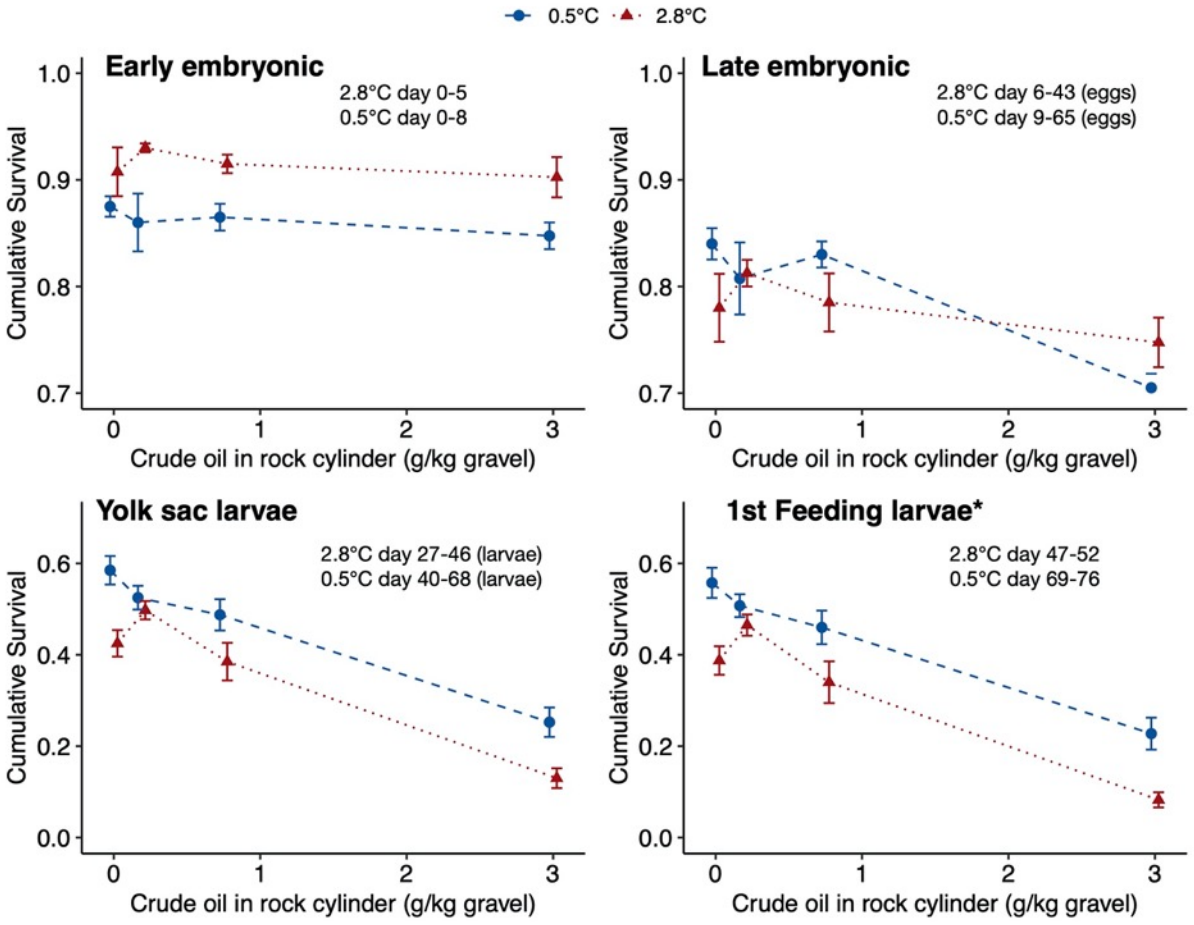

Figure S5. Cumulative survival for the four developmental stages presented in this work. Treatment means are presented with ± SEM depicted as bars, each point includes 4 incubator ratios of alive/total embryos and larvae. Colors, symbols, and line type distinguish the temperature groups. Developmental stage duration varied with temperature and is displayed in the top right of each panel. Each incubator had 9000 ± 1000 eggs at the start of the experiment.

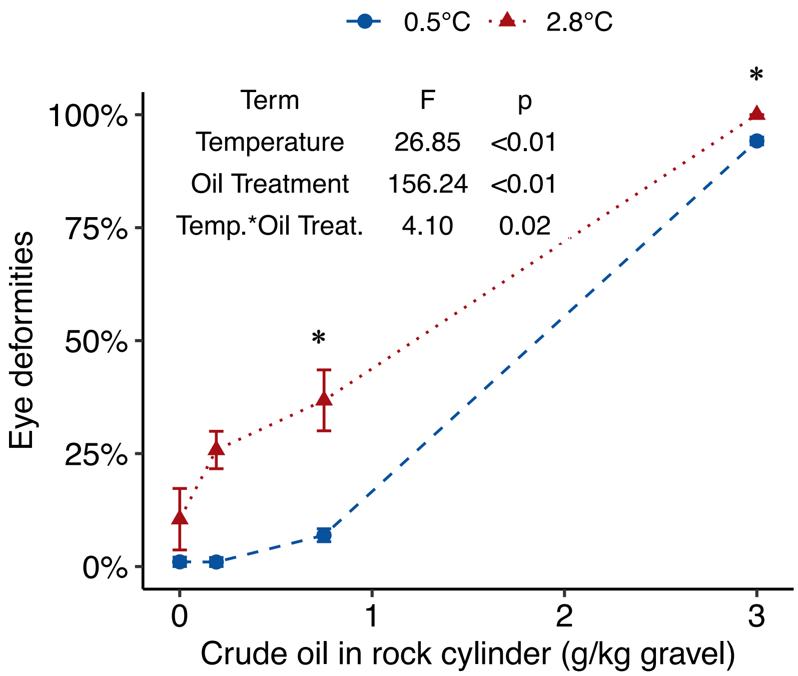

Figure S6. The effect of temperature and crude oil WSF exposure on incidence of eye deformities in the exogenous feeding larvae sampled at day 52 for 2.8°C and day 76 for 0.5°C. Treatment means (± SEM depicted as bars, each point includes 4 incubator scores, each incubator comprised of 30 larvae) are overlaid on fitted lines produced from the lme model. Model outputs and p-values are displayed in the upper left of the panel. Colors, symbols, and line type distinguish the temperature groups. The terms of the LME model for prevalence of eye deformities is displayed with the associated ANOVA test F-values and p-values. An asterisk (*) above a treatment group indicates a statistically significant difference from the unexposed group.

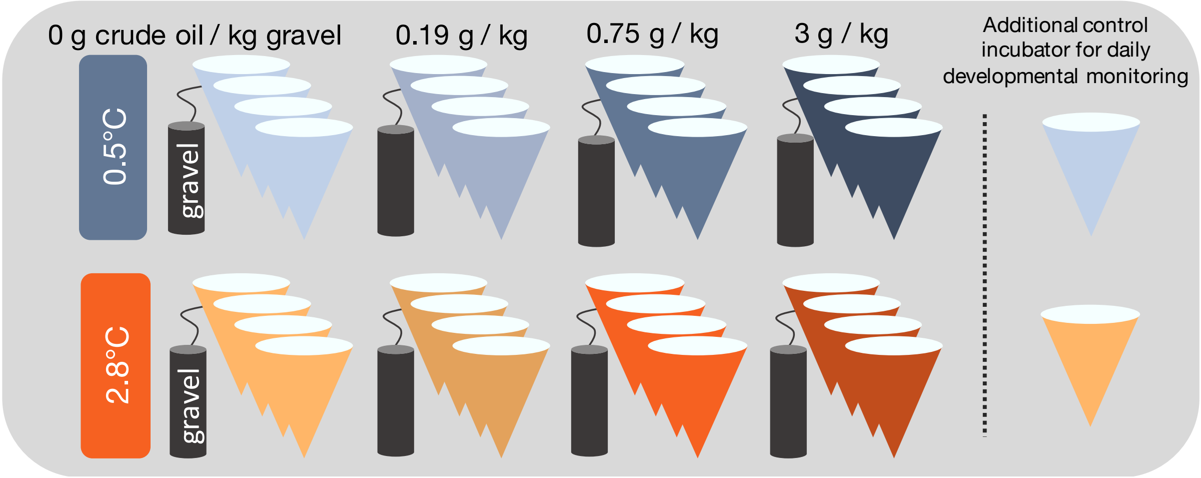

Figure S7. Experimental design with temperature treatments and crude oil exposure levels in groups with four replicate incubators, each incubator contains 9000 ± 1000 eggs at the start of the experiment.
